# Self‐Warning and Self‐Repairing Mechanisms in Functional Coatings: A Review

**DOI:** 10.1002/EXP.20240066

**Published:** 2025-03-07

**Authors:** Yixin Chen, Qian Wang, Lifeng Hou, Hao Huang, Zhiqiang Gao, Yinghui Wei

**Affiliations:** ^1^ College of Materials Science and Engineering Taiyuan University of Technology Taiyuan Shanxi China; ^2^ Corrosion and Protection Engineering Technology Research Center of Shanxi Province Taiyuan Shanxi China

**Keywords:** coating, corrosion, dual‐ functional, self‐repairing, self‐warning

## Abstract

Coatings have attracted widespread attention in the field of corrosion protection of metals because of their corrosion resistance and convenient techniques. Unfortunately, till now, traditional coatings have the shortcomings of vulnerability and passive corrosion protection, hence functional coatings progressively replace them. Endowing coatings with additional functions not only transform them into active protection mechanisms but also significantly improve life cycle of coatings. However, there is only limited success in combining multiple functions of coatings, which poses considerable obstacles to further advancement of their application researches. In this paper, we summarize the research progress of self‐warning and self‐repairing coatings in the field of metal corrosion protection as much as possible from the perspective of functional material selection. Meanwhile, the current progress of substituting dual‐functional coatings for single‐functional coatings is also highlighted. We aim to provide more options and strategic guidance for the design and fabrication of functional coatings on metal surfaces and to explore the possibilities of these designs in practical applications. Last but not least, the remaining challenges and future growth regarding this field are also outlined at the end. It is hope that such an elaborately organized review will benefit the readers interested to foster more possibilities in the future.

## Graphical abstract

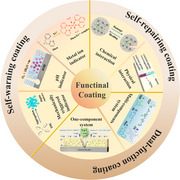

## 1 Introduction

Corrosion significantly impacts the operational reliability, safety, and lifespan of metals in sectors such as construction, transportation, and aerospace [1]. Consequently, corrosion protection is a pivotal issue, not only for economic and safety considerations, but also for its potential to extend the lifespan of products and structures, consequently leading to reduce expenditure of resource. For decades, a range of protection systems have been exploited, including alloying treatments [2], cathodic protection [3], corrosion inhibitors [4], and protective coatings [5]. In the midst of these methods, protective coatings are the most prevalent methods for safeguarding metal surfaces, which can effectively shield metals from corrosive media because of their cost‐efficiency, straightforward preparation, and suitability for application over large areas [6, 7]. In recent researches, coatings have been advanced beyond mere barrier functions, incorporating additional functional properties, not limited to superhydrophobic [8, 9], self‐cleaning [10, 11], anti‐freezing [12, 13], and anti‐fogging [14, 15].

**FIGURE 1:**
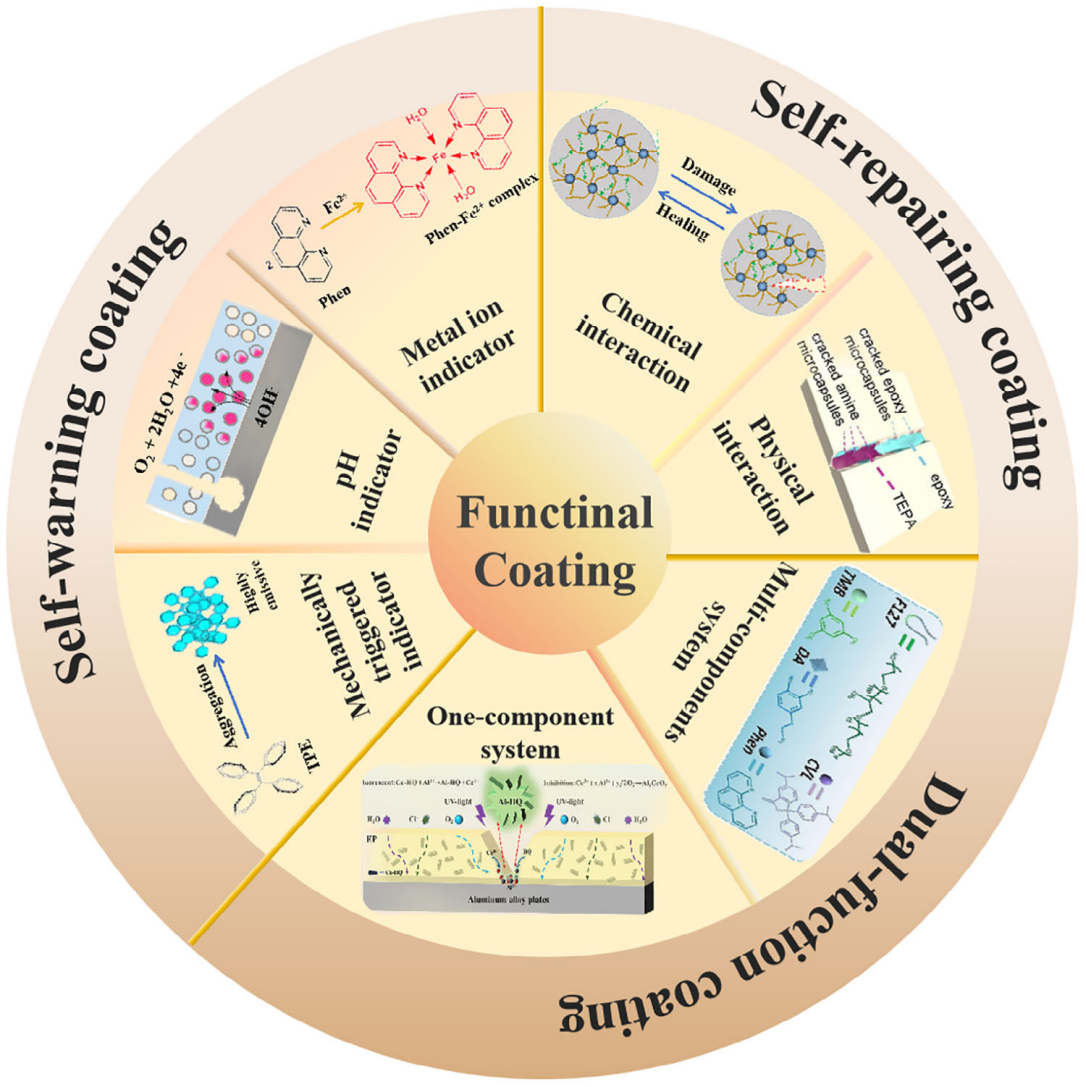
Schematic diagram of categories associated with self‐warning coating, self‐repairing coating, and dual‐functional coating.

However, damages and cracks inevitably occur in the coatings during operational services, thereby considerably undermining their anti‐corrosion performance and lifespan. Therefore, it is an urgent requirement to inspect coating damages and metal corrosion at early stages, allowing for timely intervention before the onset of severe metal degradation. Previously, some equipments have been utilized to assess the integrity of both coating and underlying substrate, such as electrochemical measurements [16], thermal imaging [17], ultrasonic [18], and acoustic emission [19]. Unrealistically, these conventional inspection methods rely on large‐scale equipment and are limited to detect damages over large and hidden regions. Self‐warning function empower the coatings to detect macroscopic damages and even nanoscale cracks by themselves. Besides, self‐repairing function is another indispensable feature of the coatings, which can repair damages and restore barrier performance. In recent years, the exploitation of dual‐functional coatings with self‐warning and self‐repairing properties has emerged as an emerging and promising frontier in the field of corrosion protection research (Figure 1).

Self‐warning coatings can warn of coating damages and metal corrosion in time, enabling early manual intervention to restore protective properties before metal undergoes further aggressive corrosion. Corrosion typically involving two parallel reactions: anodic reaction in the metal undergoes oxidation into ions; cathodic reaction in O_2_/H_2_O from environment undergoes reduction to form hydroxide ion [20, 21]. Both reactions can supply stimulative sources for corrosion indicators, including pH variation and increase of metal cation concentrations, which can make indicators show active fluorescent signals or color changes within local defects. Especially in aqueous environment, corrosion initiation process elevates the concentration of metal ions leached in the anodic zone and OH^−^ on the cathode surface. In order to track the corrosion micro‐evolution mechanism and identify active locations before corrosion products are visualized, these indicators are generally to be encapsulated, which can provide physical isolation from coating matrix and the stimulation of triggered release [22].

However, coating defects inevitably serve as diffusion pathways for corrosion medium [23, 24], implying that the onset of substrate corrosion signifies mechanical impairment of the coating. In case of mechanical damage to the coatings, mechanically triggered indicators can be released from scattered capsules, react directly with coating components and exhibit fluorescence or color changes to warn of coating damages. While these mechanically‐triggered indicators are not directly associated with metal corrosion process, they can accurately warn of coating defects, thereby indicating possible corrosion areas on metal substrates in a timely manner.

**FIGURE 2:**
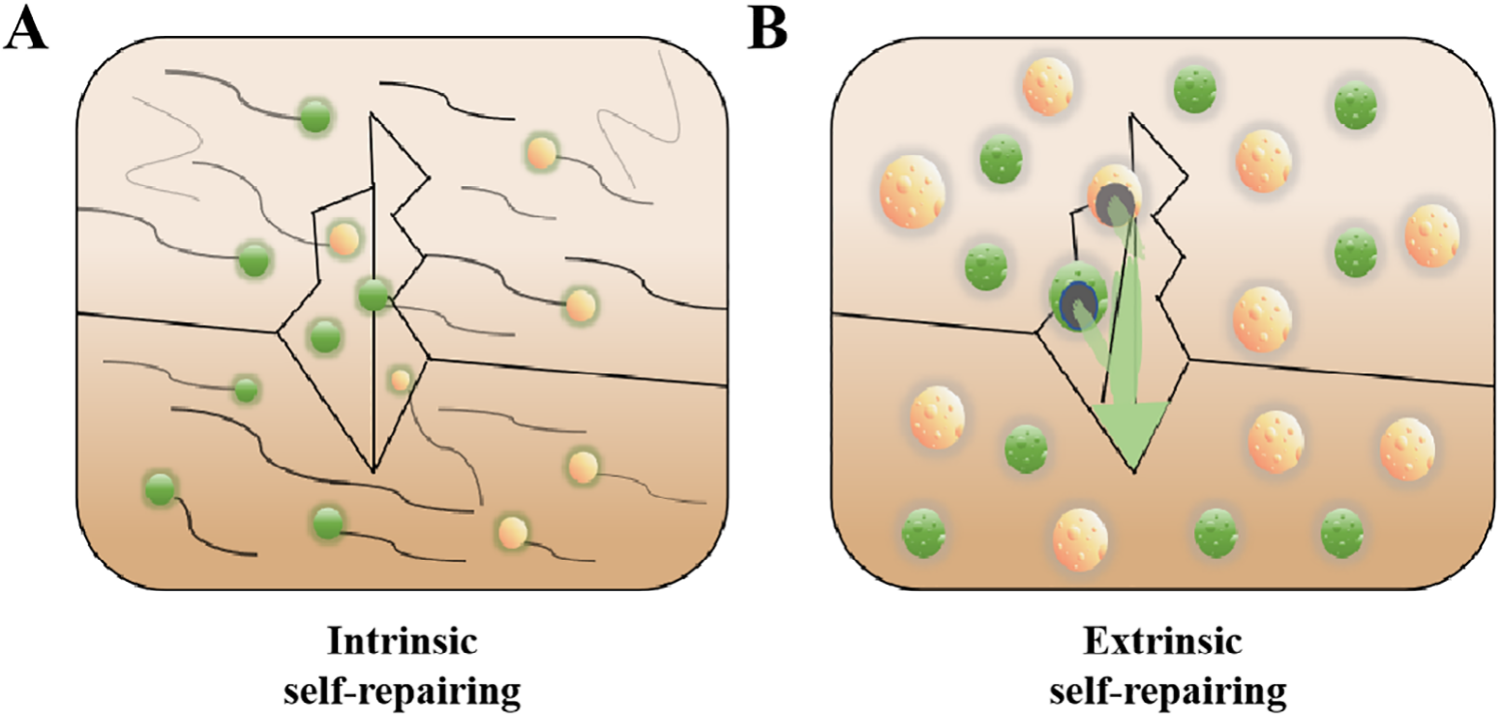
Types of self‐repairing coating. (A) Intrinsic self‐repairing and (B) extrinsic self‐repairing.

Drawing inspiration from self‐repairing ability capabilities inherent to biological species, similar properties also has been ascribed to corrosion protective coatings, enabling autonomous repair. When coatings send out warning signals of damages or corrosion initiation, they can consciously repair original performance at both macroscopic and microscopic scales. Figure 2 illustrate that self‐repairing coatings can be dichotomized into intrinsic and extrinsic categories, based on different operation mechanisms. Intrinsic self‐repairing coatings mainly capitalize on reversible covalent or non‐covalent bonds between polymer chains in coating matrix and happen cross‐linking of the polymer networks through physical or chemical reactions at the damaged sites. Unlike polymer matrices, micro/nano containers containing corrosion inhibitors/healing agents become chief architects to construct extrinsic self‐repairing coatings [25], these agents engage in reactions with either catalysts in coatings or with environmental moisture/oxygen and polymerize into thin films that fill the coating defects. Micro/nano containers not only respond quickly to stimulus release, including corrosive ions, pH changes, mechanical damages and light [26, 27], but also have superior physical characteristics with small size and large encapsulation space, which can significantly enhance the potential applicability and effectiveness of self‐repairing coatings [28, 29].

**FIGURE 3:**
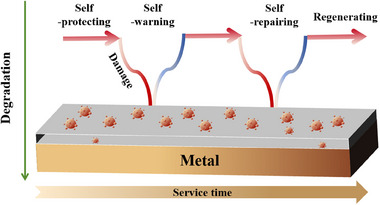
The diagram of coating service‐life cycle.

Currently, some reviews focus on aspects such as the preparation of smart‐responsive micro/nano containers, the selection of self‐repairing ingredients, polymer matrices and other functional coatings [30, 31]. Admittedly, these articles provide design insights and technical guidance for researchers. However, self‐warning and self‐repairing coatings have not been methodologically discussed and timely summarized, the important role of autonomous functions during coatings service life has been neglected. As illustrated in Figure 3, the combination of self‐warning and self‐repairing functionalities empowers coatings to maintain and restore protective properties throughout its life cycle, just like biological organisms. Concisely, when cracks or other forms of damages occur in coatings during service, self‐warning function can provide early warning and indicate the onset of coating deterioration or corrosion propagation. Meanwhile, self‐repairing ability can also be activated, allowing the coating system to mitigate further corrosion and recover initial structure. Ideally, the cycle of self‐protecting — self‐warning — self‐repairing in dual‐functional coating systems can go through many cycles, significantly bolstering the resistance of coating systems against external environmental damages. However, the integration of dual‐functional coatings have not yet matured, we categorize them into two categories based on their compositional elements: single‐component systems and multi‐component systems.

Thus, it is necessary to synthesize the preparation of self‐repairing coatings, self‐warning coatings, and dual‐functional coatings, as well as the selection of functional materials. This will provide an in‐depth understanding of the rational design of future functional coatings. The latest research advancements in dual‐functional coatings is systematically classified and summarized to explore further possibilities for future development.

In this review, we focus on dual‐functional coatings on the basis of summarizing the research advancements of self‐warning coatings and self‐repairing coatings. Indicators in self‐warning coatings are classified into metal ion indicators, pH indicators, and mechanically triggered indicators based on the sources of color/fluorescence stimulus. The description of indicators is organized around these aspects, including the diverse self‐warning strategies, the selection of indicators, the early warning effectiveness and the broader implementation of self‐warning coatings. In the discussion of self‐repairing coatings, we dissect the mechanisms underpinning both intrinsic and extrinsic self‐repair processes. The intrinsic approach focuses on chemical and physical interactions facilitating self‐repair, whereas the extrinsic methodology emphasizes the role of micro‐encapsulated and diverse stimulus‐responsive micro/nano containers in effectuating coating restoration. Furthermore, dual‐functional coatings are bifurcated into single‐component and multi‐component systems, reflecting their compositional diversity. It is hope that this review can provide prospects on the critical obligations and challenges, aiming to facilitate more research endeavors and expand application possibilities of organism‐like functional coatings.

## 2 Challenges Encountered by Advanced Functional Coatings

In the advancement of self‐warning and self‐repairing coating technologies, the integration of additional materials is crucial for realizing their functionalities. Self‐warning coatings utilize corrosion indicators or mechanically triggered indicators to warn of early corrosion or coating damages. Concurrently, self‐repairing coatings are categorized into two distinct types based on their operational mechanisms: extrinsic and intrinsic. Extrinsic coatings facilitate self‐repair by releasing corrosion inhibitors from micro/nano containers. Nonetheless, a significant challenge in fabricating these intelligent coatings lies in preserving the efficacy of materials. Once the embedded functional components are depleted, their utility becomes transient. Moreover, the incompatibility between functional agents and polymer matrix of the coatings, coupled with the possibility of chemical interactions between them, might cause the unregulated discharge of functional agents, which can undermine the overall integrity of coatings.

### 2.1 Safeguarding Functional Materials Effectiveness

In recent years, functional coatings based on micro/nano containers have been innovatively designed as a cutting‐edge anti‐corrosion technology. Within these coatings, functional components are preloaded into micro/nano containers, and then uniformly distributed in the polymer matrix. These containers not only offer a protective haven for the encapsulated active substances, acting as gatekeepers, but also are capable of sensitively detecting environmental changes caused by corrosion and responding swiftly. As a result, once triggered, the encapsulated active substances are released, unleashing their functional effects. This not only signals an early warning against the initial signs of corrosion but also facilitates further repair of the coating, significantly extending its service life. Such systems designed to functionalize surface coatings on metallic substrates have gradually become a focal point of research. Various of micro/nano containers have been ingeniously designed to provide superior service for self‐warning and self‐repairing coatings.

For micro/nano containers loaded with active substances, they must satisfy several key performance metrics, including size control, loading efficiency, and controlled release capabilities, where challenges remain formidable. Micro/nano containers with high loading capacity ensure an adequate supply of functional materials, such as corrosion indicators and inhibitors. Upon mechanical forces causing crack propagation in coatings, these containers will rupture and release functional materials at defect sites, facilitating early warning or repair functions. Thus, the size of the micro‐scale containers needs to be controlled within the range of 80–200 mm to ensure sufficient storage space for the required amount of functional materials.

Microcapsules, as common organic micro‐scale structural containers, are renowned for their exceptional loading capacity. Their capsule walls are designed to enable rupture and release upon mechanical damages, making them suitable for encapsulating flowable liquid functional materials. However, these organic containers face a significant challenge: their complex fabrication process, which involves polymerization reactions, reagent encapsulation, and the removal of by‐products and solvents [32]. Conversely, nanocontainers are more suitable for encapsulating solid functional materials. Mesoporous silica, for example, is favored for its small size, high stability, extensive surface area, and straightforward encapsulation process. Its main limitation lies in compatibility issues with the polymer matrix, which may lead to aggregation phenomena, thus deteriorating the performance of coatings.

In recent research, compatibility between functional agents and polymer matrices has been a challenging issue. Many methods that directly disperse the agents into resin face problems like poor probe stability, easy dissolution from the resin and short coating lifespan. The compatibility between various agents and coatings directly determines whether the method can successfully achieve corrosion sensing. To overcome these limitations, chemically grafting the agents onto the resin shows better performance than directly dispersing reagent molecules into the coating. However, due to the challenges of the chemical grafting process, using micro/nano containers is a viable alternative solution, isolating the indicator from coating components to resolve compatibility issues. Micro/nano containers also offer additional benefits of insulation and improved sensing sensitivity of the coating.

Thus, it is critical to select suitable containers for functional materials when designing self‐warning or self‐repairing coatings. This requires not only good interfacial compatibility between the containers and the polymer matrix, but also desirable properties of the containers themselves. These properties include straightforward fabrication methods, efficient loading capacities, and precise controlled‐release capabilities. Tackling these issues is a pressing challenge in the field of functional coatings research.

### 2.2 Balancing Coating Performance Attributes

Coatings are targeted to offer barrier protection and additional specialized functions to metal substrates, such as corrosion resistance, self‐repairing capabilities and self‐warning properties. However, effectively integrating these diverse functions into a singular coating system while preserving the fundamental performance of the coating presents a significant challenge. Achieving excellent compatibility among multiple functionalities in coatings necessitates a holistic strategy, which involves the meticulous selection of functional materials, the design of fabrication processes and the characterization of individual properties.

Ensuring the harmonious integration of diverse functions in a single coating system requires a strategic approach, beginning with the selection of materials. The key is to identify materials that either inherently have, or can be chemically modified to exhibit, the multiple desired properties. For instance, intrinsic self‐repairing coatings require sufficient elasticity and fluidity to ensure rapid and effective recovery after damage. Moreover, it is essential to ensure that these characteristics do not adversely affect the other properties of the coating. Secondly, the optimization of chemical composition is key to attaining a balance between multi‐functionality and performance. By precisely controlling the reaction conditions and the quantity of additives, it is possible to regulate the interaction between the different components of the coating. This allows certain functionalities to be maintained while optimizing other performance. Furthermore, the parameters of fabrication process play a significant role in maintaining the balance of coating performance. The preparation conditions of coatings, such as temperature, duration and curing methods, must be precisely designed and controlled to ensure the uniform distribution of different functional components, thereby avoiding performance degradation due to uneven dispersion. Last but not least, the practical application environment of coatings must also be taken into account. Factors such as the stability, durability and responsiveness of the coating functionalities in specific environments are crucial to ensure that coatings achieve the intended effects in practical applications.

Moreover, functional coatings face multiple challenges in terms of cost‐effectiveness and scalability, therefore it is essential to optimize the synthesis and application processes to minimize resource utilization and waste generation while achieving the requisite coating functionalities. The incorporation of advanced functions into coatings inherently involves complex materials and methods, which may raise costs and complicate scalability. Therefore, tackling these challenges is crucial for facilitating the transition of functional coatings from research and development stage to broad commercial applications, underscoring the need for innovative approaches to reconcile performance with economic and production feasibility in this evolving field.

## 3 Classification of Indicators for Self‐Warning Coatings

Corrosion normally involves two parallel reactions: Equation (1) anodic reaction (oxidation of metal); Equation (2) cathodic reaction (reduction of oxygen or water molecules in environment):

(1) $$\mathrm{Anodic}\;\;\mathrm{reaction}:\;M\to ne^{-}+M^{n+}$$

(2) $$\mathrm{Cathodic}\mathrm{reaction}:\;O_{2}+2H_{2}O+4e^{-}\to 4{\mathrm{OH}}^{-}$$

The reactions can result in the generation of metal ions in the anodic zone and pH changes in the cathodic zone with the accumulation of hydroxide ions [21]. Corrosion at early stages will come into being two sources for activating indicators: elevated concentrations of metal cations and pH variations, both of which can alter the fluorescence/colors of indicators by changing molecular structures or complexing with metal ions to yield color‐coded compounds. On top of that, certain indicators can directly interact with coating components, thereby unveiling mechanical damages. In spite of the fact that mechanically triggered indicators are not directly linked to corrosion process, they can supply precise indication of coating damages and warn of potential sub‐film corrosion.

### 3.1 Corrosion‐Related Indicators for Metal Ions

Molecules endowed with corrosion warning capabilities can exhibit significant fluorescence enhancement or color changes through complexation with special metal cations generated from corrosion. The luminescence properties of fluorescent probes have been researched, for example, Sun et al. [33] reviewed the different types of fluorescence emission mechanisms exhibited by coumarin derivatives. The choice of metal ion indicator should firstly consider the category of substrate and whether it complexes with the indicator. Subsequently, warning signals should be considered to exhibit fluorescence or color change visible to the naked eye under white or UV light. Coatings designed to alter color or emit fluorescence upon the onset of corrosion can significantly streamline the visual inspection process. In this section, mainly focusing on the utilization of Fe^2+^/Fe^3+^ indicators, Al^3+^ indicators and Cu^2+^ indicators. Table 1 lists the metal corrosion or coating damage indicators and their applications in self‐warning coatings.

**TABLE 1:** Metal corrosion or coating damage indicators and their applications in self‐warning coatings.

Type	Indicator	Container	Coating	Reacting specie	Reference No.
Metal ions indicators	1,10‐phenanthroline	Silica nanoparticles	Epoxy resin	Fe^2+^	[34]
	8‐hydroxyquinoline	−	Epoxy resin	Fe^3+^	[35]
	FD1	−	Epoxy resin	Fe^3+^	[36]
	Phenylfluorone	−	Acrylic coating	Al^3+^	[37]
	Rhodamine B	MOFs	Epoxy resin	Al^3+^	[38]
	8‐hydroxyquinoline	porous micro‐spheres	Epoxy acrylate	Al^3+^	[39]
	RHS	ZIF‐8	Epoxy resin	Cu^2+^	[40]
	Rhodamine	−	Epoxy resin	Cu^2+^	[41]
pH indicators	FD1	−	Epoxy resin	H^+^	[42]
	Coumarin 120	−	Epoxy resin	H^+^	[43]
	Phenolphthalein	Silica nanoparticles	Acrylic urethane	OH^−^	[44]
	Phenolphthalein	PDVB‐microcapsules	Acrylic coating	OH^−^	[45]
Mechanical trigger indicators	TPE	Microcapsules	DSRTET	−	[46]
	BPF, HPS, TPE	Microcapsules	Epoxy resin		[47]
	CVL	Microcapsules	PDMS	−	[48]

#### 3.1.1 Corrosion Indicators Sensitive to Fe^2+^/Fe^3+^

Fe^2+^/Fe^3+^ as natural product in the anodic zone of corrosion on steel substrates, so they can primordially act as stimulus source for the indicators. The ideal targets should have apparent “color transformation” mechanisms when corrosion attack, that means the warning signals can be directly observed when they shift from absence to presence. The only current indicators that complexes with ferrous ions for color‐rendering effects are phenanthroline and its derivatives. There are alternate indicators that complexes with ferric ions emit fluorescent signals under UV light, including 8‐hydroxyquinoline (8‐HQ), [1H‐isoindole‐1,9′‐[9H]xanthen]‐3(2H)‐one,3′,6′‐bis(diethylamino)‐2‐[(1‐methylethylidene)amino] (“FD1”), Rhodamine B (RhB) and its derivative.

**FIGURE 4:**
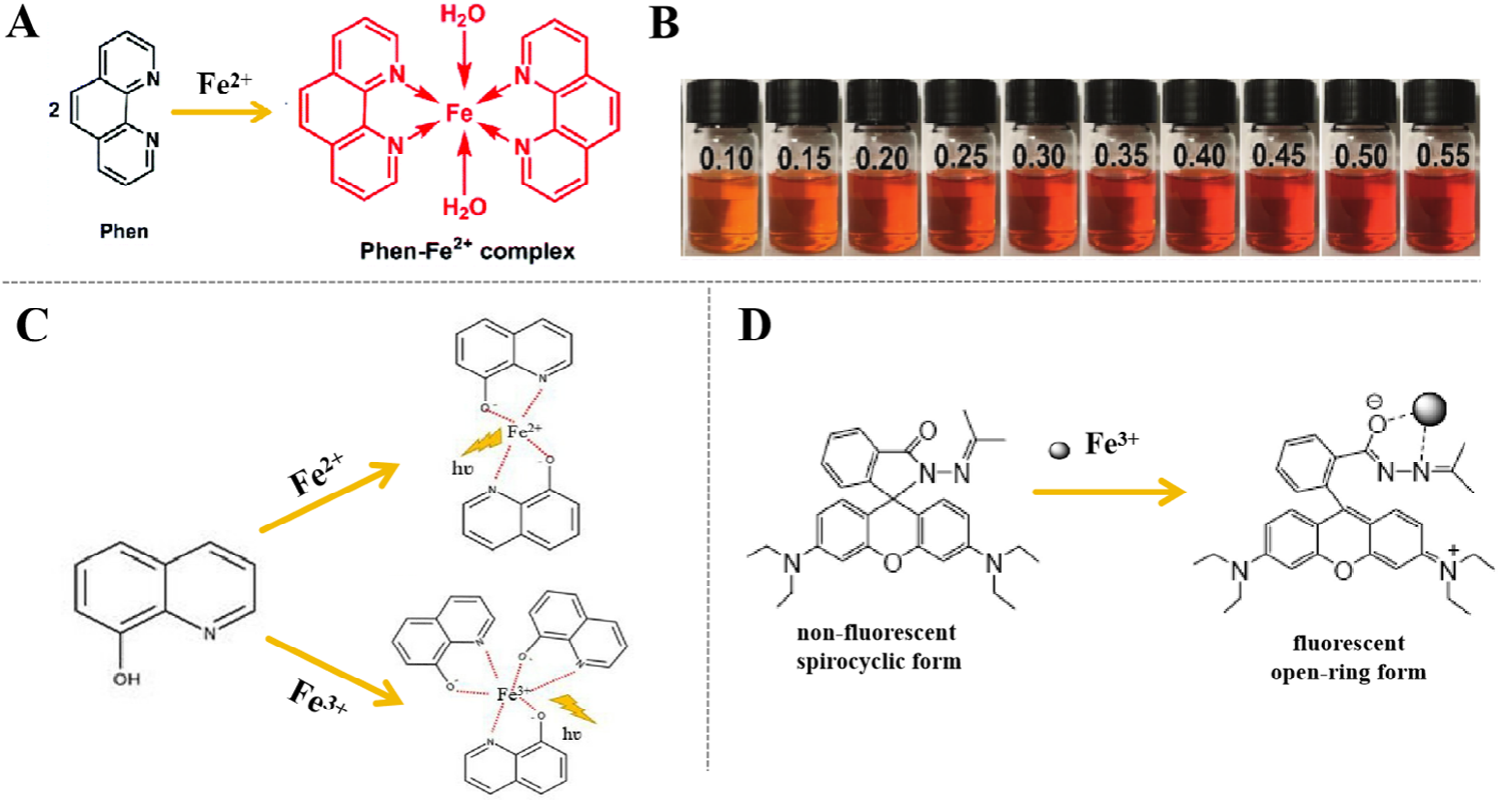
Indicators sensitive to Fe^2+^/Fe^3+^ ions. (A) The complex formed between Phen and Fe^2+^. Reproduced with permission [49]. Copyright 2020, Royal Society of Chemistry. (B) Photographs (the number indicates molar ratio of Fe^2+^ to Phen) of Phen with the addition of different concentrations of Fe^2+^ ions. Reproduced with permission [49]. Copyright 2020, Royal Society of Chemistry. (C) Turn‐on fluorescence mechanism for 8‐HQ. Reproduced with permission [35]. Copyright 2021, Taylor & Francis. (D) CHEF of FD1 upon binding with Fe^3+^. Reproduced with permission [36]. Copyright 2009, American Chemical Society.

Phenanthroline and its derivatives form bonding complexes [Fe(Phen)_2_]^2+^ with ferrous ions, which can show remarkable orange‐red color (Figure 4A). The color and concentration gradient of Fe^2+^ also expressed a relationship that there is the highest color intensity of orange when the molar ratio of Fe^2+^ to Phen is 0.5. As Fe^2+^ concentration increase, the intensity of complication color no longer increase (Figure 4B). The experimental results show that phenanthroline and its derivatives can be used as color indicator for self‐warning coatings on steel substrate [49]. Some early works performed by Dhole, who used Phenanthroline derivatives for chemical modification of acrylic polymers and alkyd resins respectively. The optimum content of 5‐acrylamido‐1,10‐phenanthroline was fixed at 2.5% and alkyd resin to 1,10‐phenanthroline‐5‐amine in modified alkyd resin was obtained as 100:1.90. The color change points formed by the two substances with Fe^2+^ are corrosion sites of steel [34, 50]. Compared with blank resin coating, the modified chemical resin coating shows superior anti‐corrosion properties, this provides us with a brilliant design conceptualization.

8‐HQ has the capability of forming fluorescent complexes with ferric ions and it is extremely hypersensitive to the ions. It's noteworthy that the presence of extra particles may lead to a decrease in the fluorescence signal of 8‐HQ‐Fe^3+^ (Figure 4C). Roshan et al. [51] used fluorescence microscopy to observe fluorescence “turn‐on” of 8‐HQ by forming complexes with Fe^3+^. Additionally, a low level of 8‐HQ (0.1 wt%) were used in the test to detect early steel corrosion, demonstrating ultimate sensitivity of 8‐HQ to Fe^3+^. As well, the warning signals should also have properties of not being interfered by the coating matrix. It has been demonstrated that the coating didn't show premature fluorescence either, when 0.1 wt% 8‐HQ was added to 2 wt% nanoclay [35]. The coatings showed both optimal corrosion resistance and highly target‐oriented targeting of corrosion product ions.

“FD1” also doesn't show premature fluorescence “turn‐on” during curing with other coating substrates (epoxy coatings), so it is also versatile in many coating systems (Figure 4D). Specifically, Augustyniak et al. [36] explained the successful application of “FD1” as the indicator in epoxy‐based coatings for early warning of steel corrosion. Earlier yellow fluorescence can be observed under UV light compared to natural light conditions. We can select different light sources to observe warning signals, which can minimize corrosion losses. Premature leakage of indicators should also be a matter of concern in preparation of coatings. In order to prevent similar phenomena, subsequent works studied by Exbrayat prepared hydrophobic silica nanocapsules encapsulating Rhodamine B derivative by microemulsion method, where Fe^3+^ can diffuse into the interior through silica shells and produce strong early warning signals due to fluorescence “turn on” [52]. Various corrosion indicators show fluorescent signals or color changes because of successful complexation with Fe^2+^/Fe^3+^, which can be moved or loaded into micro/nano containers to prepare coatings with self‐warning function. In other words, further researches are required to enhance warning efficiency and fluorescence/color endurance, so that self‐warning coatings have long‐term stability.

#### 3.1.2 Corrosion Indicators Sensitive to Al^3+^

Aluminum ions don't have a specific indicator that complexes with them to form visible color with the naked eye. With regard to the universality of the indicators that emit fluorescent signals, self‐warning coatings on the surface of aluminum substrates also partially repeat the selection of ferric ion indicators mentioned above, such as RhB, 8‐HQ. Due to protons transform from hydroxyl group to nitrogen atom within the excited state molecule of 8‐HQ, causing complexes of 8‐HQ to exhibit fluorescent source [53]. This is all based on the chelation‐enhanced fluorescence by metal cations complexed with indicator fluorophores, it's worth noting that Al^3+^ and Fe^3+^ have comparable effects.

**FIGURE 5:**
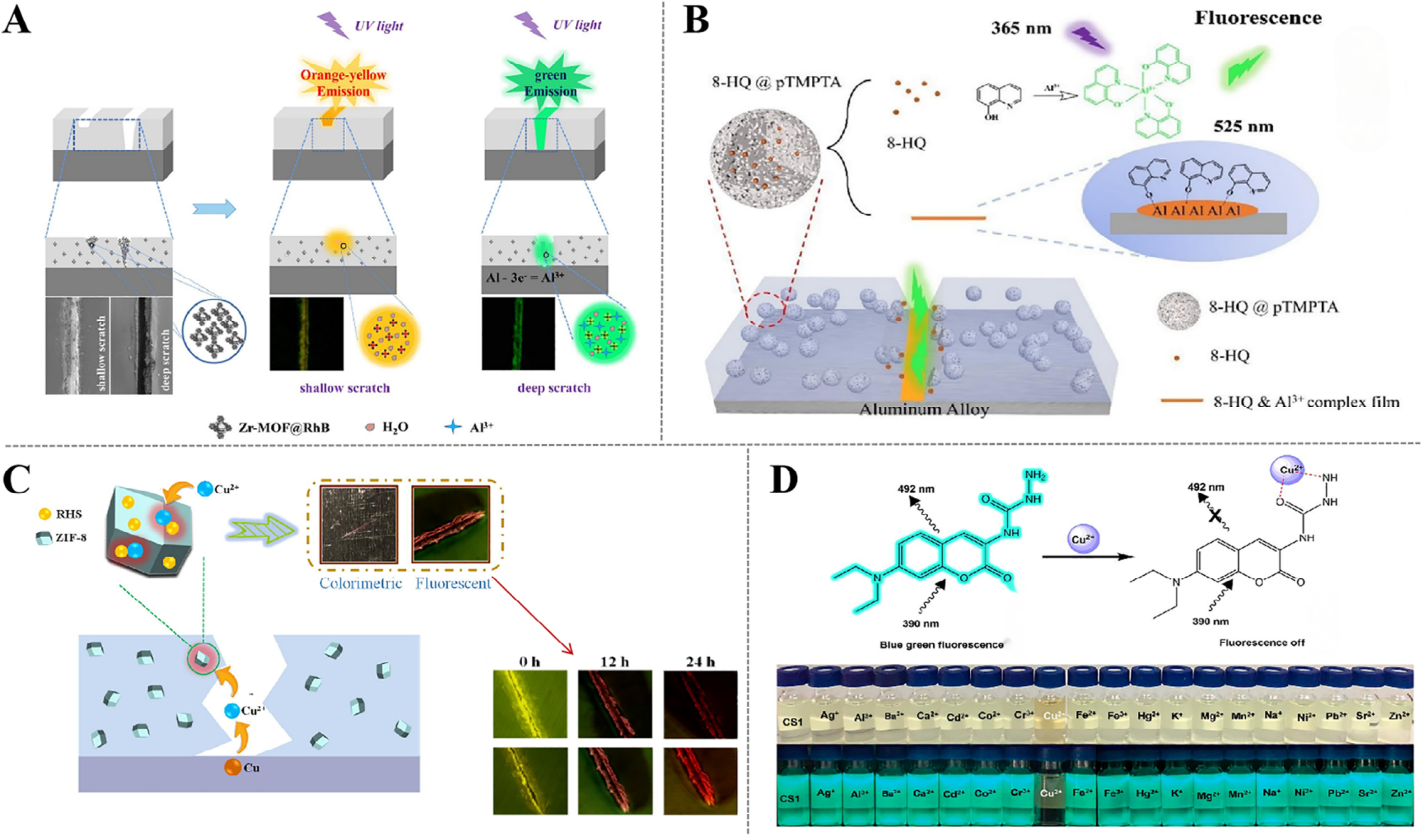
Utilization of Al^3+^ and Cu^2+^ sensitive indicators in coating applications. (A) Schematic diagram of the warning system to autonomously detect and distinguish the damage of surface coating (shallow scratch) and the corrosion of substrate aluminum (deep scratch). Reproduced with permission [38]. Copyright 2022, Elsevier. (B) Schematic diagram of self‐reporting and anti‐corrosion mechanism of coating with 8‐HQ@pTMPTA microspheres. Reproduced with permission [39]. Copyright 2023, Elsevier. (C) Schematic diagram of possible complexation mechanism of rhodamine‐ethylenediamine with copper ions. Reproduced with permission [40]. Copyright 2020, Elsevier. (D) Proposed sensing mechanism of Cu^2+^ ions by CS1 and optical change of CS1 upon addition of metal ions (20 equiv.). Reproduced with permission [62]. Copyright 2022, Elsevier.

The emission behavior of RhB composite at 490 nm is greatly enhanced with the presence of Al^3+^ ions. Recent studies conducted by Fan et al. loaded RhB fluorescent composites into metal‐organic framework (MOF) and mixed in epoxy resin coating. The self‐warning coating could recognizably detect the damage regions, in coating parts or even on aluminum matrix surface. When faint damages on the coating parts rather than metal surface, orange fluorescence could be distinguished around the areas of coating cracks. If corrosion attack the upper metal matrix, the fluorescence around scratch turns to green (Figure 5A). In short, this visual distinction response system can be a promising strategy for smart self‐warning coatings, which can identify damaged areas by radiating completely separate color signals [38].

8‐HQ is mentioned as corrosion indicator for Fe^3+^ in the previous section, but it is tested on Al^3+^ as well. In recent researches, Li prepared porous micro‐spheres loaded with 8‐HQ by facile one‐pot photopolymerization. As shown in Figure 5B, corrosive medium passed through coating damage sites to reach surface of aluminum matrix, generating Al^3+^ in anodic region to form acidic regions so that accelerating the release of 8‐HQ from the porous micro‐spheres. The vibrant fluorescence generated from 8‐HQ‐Al^3+^ complexes emit a prompt warning signals of corrosion caused by mechanical damages and pitting corrosion under the coating, delivering the self‐warning function successfully [39].

More recently studies for improving the solubility, dispersion, and sensitivity of corrosion indicators in coating matrix, apart from encapsulation of containers, exceptional surfactant can be added in functional coatings. Li et al. [37] chose Triton X‐100 as the surfactant of phenyl fluorescent ketone (PF) dye to prepare an acrylic base‐fluorescent coating for aluminum alloy. Contrary to previous cases, fluorescence off caused by anodic reactions with Al^3+^ during corrosion initiation course could be observed with naked eye under hand‐held UV light. To validate the accuracy of warning signals, they also applied constant charge current for measuring the charge, which fluorescence, facilitating the visual identification of fluorescence quenching in the coating.

#### 3.1.3 Corrosion Indicators Sensitive to Cu^2+^

There are numerous methodologies to detect Cu^2+^, including inductively coupled mass spectrometer [54], atomic absorption spectroscopy [55], and other primordial methods, hardly these techniques are complicated and time‐consuming. Now, many research endeavors have shifted towards employing fluorescent probes as excellent alternatives for Cu^2+^ indicators, the probes can be coordinated with the metal ion to generate fluorescence effects [56], which can be used to monitor the evolution of metal ion concentration in the corrosion area, and meanwhile can achieve function to warn of copper substrate corrosion.

Rhodamine fluorescent probe has dominance of high fluorescence quantum yield and terrific absorption coefficients, which make it also become an ideal material for construction of Cu^2+^ fluorescent probe. An early work performed by Wang et al. [40] reported a fluorescent probe based on RhB for Cu^2+^, the mechanism of probe is based on the reversible ring‐open process between non‐fluorescent spironolactam state and fluorescent off‐domain hydrazide state triggered by coordination between Cu^2+^ and RhB hydrazine. In order to prevent erroneously interaction between fluorescent probe and coating matrix, metal‐organic framework (ZIF‐8) was applied as nanocontainer for RHS in self‐warning coating (Figure 5C), and early warning signals could still be recognized when the concentration of RHS was reduced to only 0.14 wt%. It is reported that a method to certificate early self‐warning function of rhodamine ethylenediamine on copper artifacts of epoxy coatings. Adding 0.8 wt% rhodamine ethylenediamine to epoxy coating can effectively monitor early corrosion of copper artifacts. With the prolongation of immersion time, the fluorescence intensity of corrosion areas in coating became more enhanced, the mechanism that rhodamine‐ethylenediamine was disrupted by the ring‐opening of spironolactam and Cu^2+^ to form a seven‐membered ring with conjugated structure [41].

The design and synthesis of Cu^2+^‐selective fluorescent probes are based on different signal mechanisms [57], such as light‐induced electron transfer, fluorescence‐energetic electron transfer, internal charge transfer, complexation‐induced fluorescence quenching, and paramagnetic fluorescence quenching mechanisms, etc. Many receptors have been reported, for instance, quinazoline [58], fluorescein [59], pyrene [60], coumarin [61], etc. A single fluorescent probe have stability shortcoming, and there are new design ideas of combining two fluorescent probes. Consequently, Srisuwan [62] developed a novel synthetic aminouracil derivative based on coumarin (CS1) that acted as a fluorescent probe for detection of Cu^2+^ (Figure 5D).

In the case of metal ion indicators, the more commonly used are 8‐HQ, RhB, “FD1”, etc. These indicators can match two or more types of metal ions, and some of indicators are only complexed with specific ions to change the color, such as Phen. Up to now, other ions have not been studied for color development reactions, especially Mg^2+^, it is thus of immense value to develop novel metal ion indicators. To further improve fluorescent/color durability, it is necessary to study the relationship between corrosion degree and micro‐environmental changes at coating defects, such as spatial distribution of metal ions and their evolution with time. These are not only related to types of metal substrates, but also involved in composition of coatings as well as the size and depth of defects. Most of fluorescent probes have limitation in terms of performance, such as imprecise test range, low sensitivity and limited applications, etc.

### 3.2 Corrosion‐Related Indicators for pH Changes

In the corrosive environment at the cathode, exogenous oxygen and water molecules or H^+^ from the corrosive medium access metal matrix by penetrating from the coating punctures or cracks, and derive electrons from the anode to metal surface [63, 64]. The alkalinity of localized region is progressively increased during corrosion procedure in the form of sequential dynamic changes in OH^−^ accumulation or H^+^ depletion, coexisting with an increase in pH levels. Therefore, we can visually discern the early matrix corrosion by utilizing coatings that contain pH indicators, which can produce flash color or fluorescence variations under visible or UV light in a specific pH range.

**FIGURE 6:**
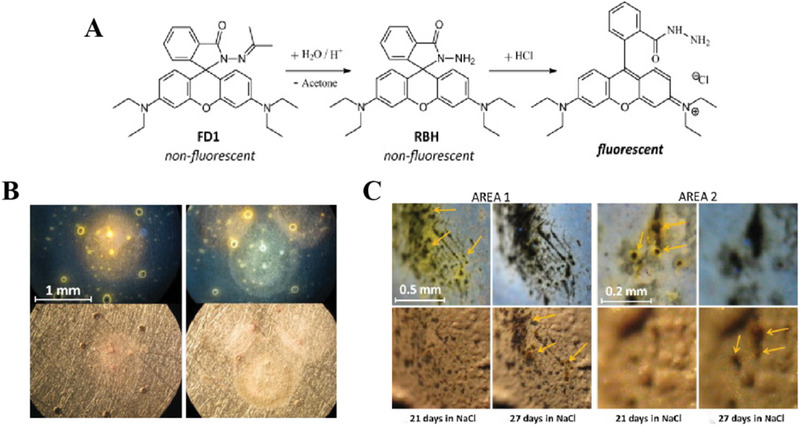
Application of low pH‐sensitive indicator in self‐warning coatings. (A) Proposed mechanism of FD1 fluorescence at low pH. (B) Images of Al 1052 coated with a FD1‐containing, clear epoxy coating after 2 days (left) and 3 days (right) of exposure to 3.5% NaCl solution. (C) Digital camera images taken through the confocal microscope eyepiece under UV light (top) and natural light (bottom). Adapted with permission.[42] Copyright 2011, Elsevier.

“FD1” molecule was initially proposed as a Fe^3+^ sensor for biological applications. It was also noted that the fluorescence of “FD1” increased significantly with decreasing pH. Spirocyclic “FD1” is sensitive to low pH due to its acid‐catalyzed hydrolysis to Rhodamine B hydrazine, then can be protonated to the fluorescent ring‐opening form (Figure 6A). For the case of chemical structure transformation, which can trigger fluorescence code to switch on. It has been reported that the successful application of spirocycle “FD1” in epoxy coatings for early warning of aluminum corrosion. Augustyniak tested that “FD1” did not form a fluorescent complex with Al^3+^, so the fluorescent emission observed was attributed to the low pH at the anodic site of aluminum corrosion, which may reach as low as 3.5. Figure 6B showed that the color and fluorescence was exceptionally luminous around air bubble defects, which was caused by the fact that corrosion occurred much faster in these flawed areas. When coating was exposed to 3.5% NaCl solution for 2month days, lively orange spots could be markedly observed in severe corrosion zones (Figure 6C). [42]

In contrast to aforementioned cases, coumarin derivatives exhibit fluorescence off under low pH conditions, at localized corrosion regions, coating defects are initially transition from “light” to “dark” representing self‐warning signals. Up to now, only Liu et al. [43] used the fluorescence off mechanism to detect aluminum corrosion by adding coumarin 120 as fluorescent indicator to coatings, which showed that the coating was initially fluorescent and became non‐fluorescent at corrosion sites under UV light irradiation. After immersion in 3.5% NaCl solution for 24 h, fluorescent state begin to weaken, that indicated the beginning of corrosion. More notably, this study also found that the fluorescence intensity gradually decrease with the increase of coating thickness, and optimal coating thickness was ∼80 µm, once again testifying the thickness of the self‐warning coatings is a prominent factor for observing corrosion signals. Strictly speaking, this mechanism has great demands on the thickness and transparency of coatings.

Hydroxide ions are formed during corrosion in aqueous environment and are central of corrosive electrochemistry. In addition to fluorescent dyes, some indicators exhibit visual color changes with pH variation. For instance, phenolphthalein (PhPh) [44, 65]. bromothymol blue [66], bromocresol green [67], and cresol red [68]. PhPh is preferred as the most satisfactory indicator for detecting hydroxide ions because of its striking color contrast during pH variegation, being colorless at pH value below 8.2 but turning to bright pink at pH range of 8.2–12.0.

**FIGURE 7:**
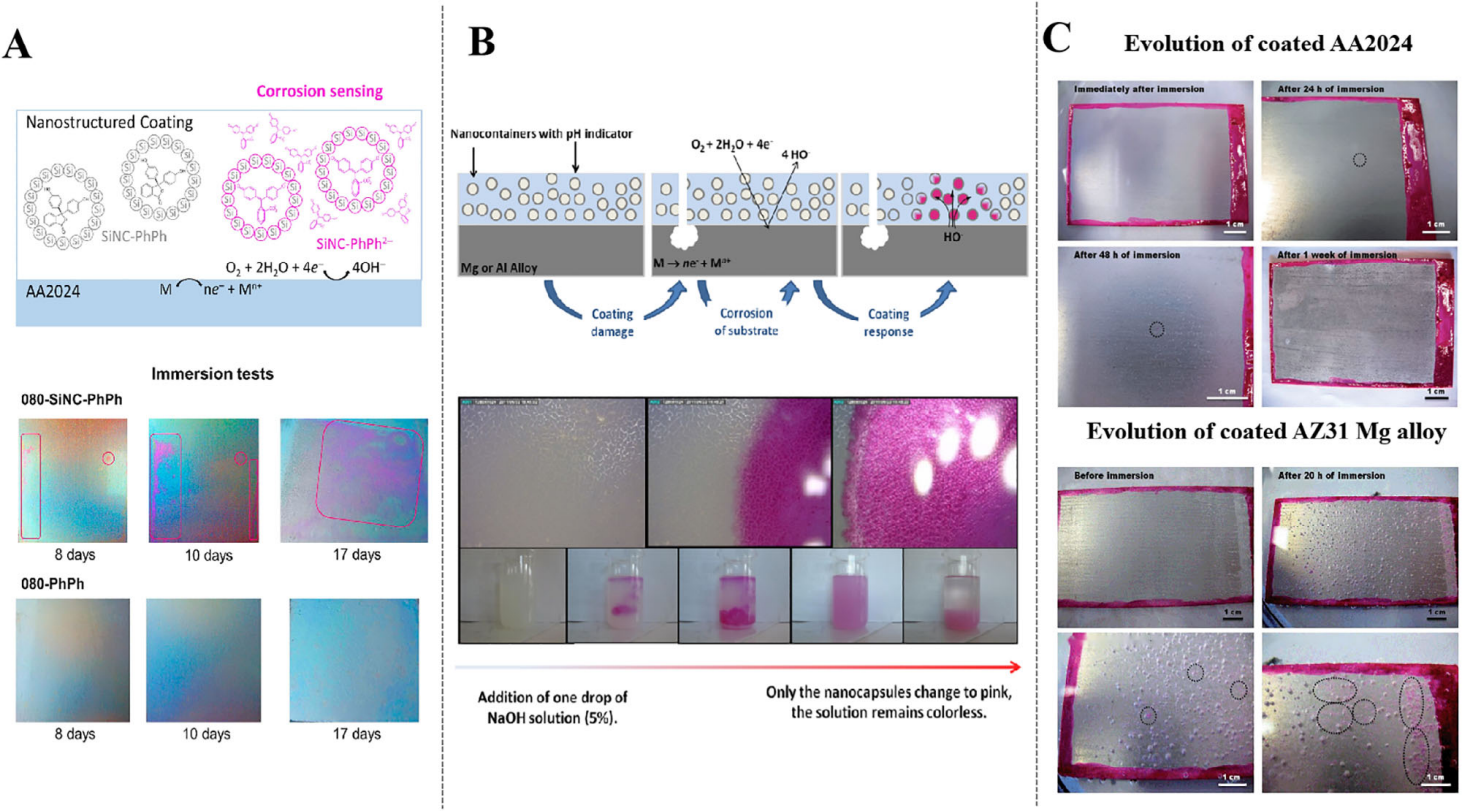
Applications of phenolphthalein (PhPh) in self‐warning coatings. (A) corrosion sensing mechanism of SiNC‐PhPh corrosion sensing coating for AA2024 and immersion tests of coated AA2024 plates in 5% NaCl. Reproduced with permission [44]. Copyright 2018, Elsevier. (B) scheme of pH sensing response from coating and color development of PhPh‐containing nanocontainers in solution. Reproduced with permission [64]. Copyright 2013, Elsevier. (C) evolution of coated AZ31 Mg alloy immersed in a 0.5 m NaCl solution. Reproduced with permission [45]. Copyright 2014, Royal Society of Chemistry.

In order to protect PhPh from premature reactions with coating substrates, many researchers have encapsulated PhPh in nanocontainers for self‐warning coatings on magnesium/aluminum alloy (Figure 7B). Recently, studies performed by Galvã [44], who incorporated silica nanocontainers encapsulated with PhPh into waterborne paints (Figure 7A), where the shell of silica will activate indicator to minimize undesirable interactions with coating matrix. Further dipping and salt spray tests, only samples encapsulated with PhPh (080‐csc‐PhPh) were able to show color changes for warning of early corrosion. Apart from macro color observations, computer simulations based on density functional theory and periodic structure models have been performed to shed light on the interaction pattern of phenolphthalein with metal surfaces. The validation study of pH‐sensing/color‐signaling functionality of SiNC‐PhPh containing PEI coatings on magnesium alloy matrix was reported. Another distinct category, which showed fast color signals matching the corrosion kinetics of metal surface by differential viewer imaging technique (DVIT)‐assisted electrochemical impedance spectroscopy (EIS) tests. These works also open new objectives for future of self‐warning coatings, which is meaningful to quantify and directly correlate change of color and its intensity with the level of degradation measured electrochemically.

There is also a necessity to do some researches on the compatibility between encapsulating containers and coating matrix. Microcapsule, serving as organic nanocontainer within coatings [44, 69], which is characterized by favorable compatibility with polymer matrices in well‐dispersed coatings. Therefore, a case used polymer (polyurea) as its wall material, due to its chemical composition is similar with microcapsule as the wall material, so that largely improve the compatibility between microcapsules and coating matrix [45]. As displayed in Figure 7C, the effects of cathodic reaction on coated AZ31 magnesium alloy are remarkably different from AA2024, because of higher degradation of magnesium alloy. It is very important to warn of early degradation stages and act in a preventive way to guarantee structural safety of materials. Regardless of silica or microencapsulated nanocontainers, we need to take into account that the key factor for self‐warning lies in the prominent color change of PhPh under alkaline conditions, the pink color of PhPh turn back to its colorless structure form once pH value exceeded 12. Hence, the addition amount and distribution of PhPh‐based need to be optimized in order to provide preferable coloring kinetics and long‐term durability.

### 3.3 Mechanically Triggered Indicators Related to Coating Damage

Apart from the fluorescence/color effects triggered by metal ions or pH changes, mechanical trigger indicators, upon release from compromised capsules, can directly engage with coating matrix or exhibit intra‐molecular movement to warn of coating damages [70]. In 2009, spiropyran emerged as a quintessential mechanochromic molecule, which is chemically integrated into the polymer matrix, and undergoes a ring‐opening reaction, transitioning from yellow to purple color in response to mechanical forces [71]. It can be seen that this indicator must be bonded to other substances and change the color by converting its chemical structure under external forces. However, alternate mechanical trigger indicators can be independently encapsulated in stimulus‐responsive micro/nano containers. Aggregation‐induced emission (AIE) chemicals and crystal violet (CVL) represent the more widely adopted mechanically triggered indicators in self‐warning coatings.

#### 3.3.1 Fluorescence Signals of AIE for Damage Detection

**FIGURE 8:**
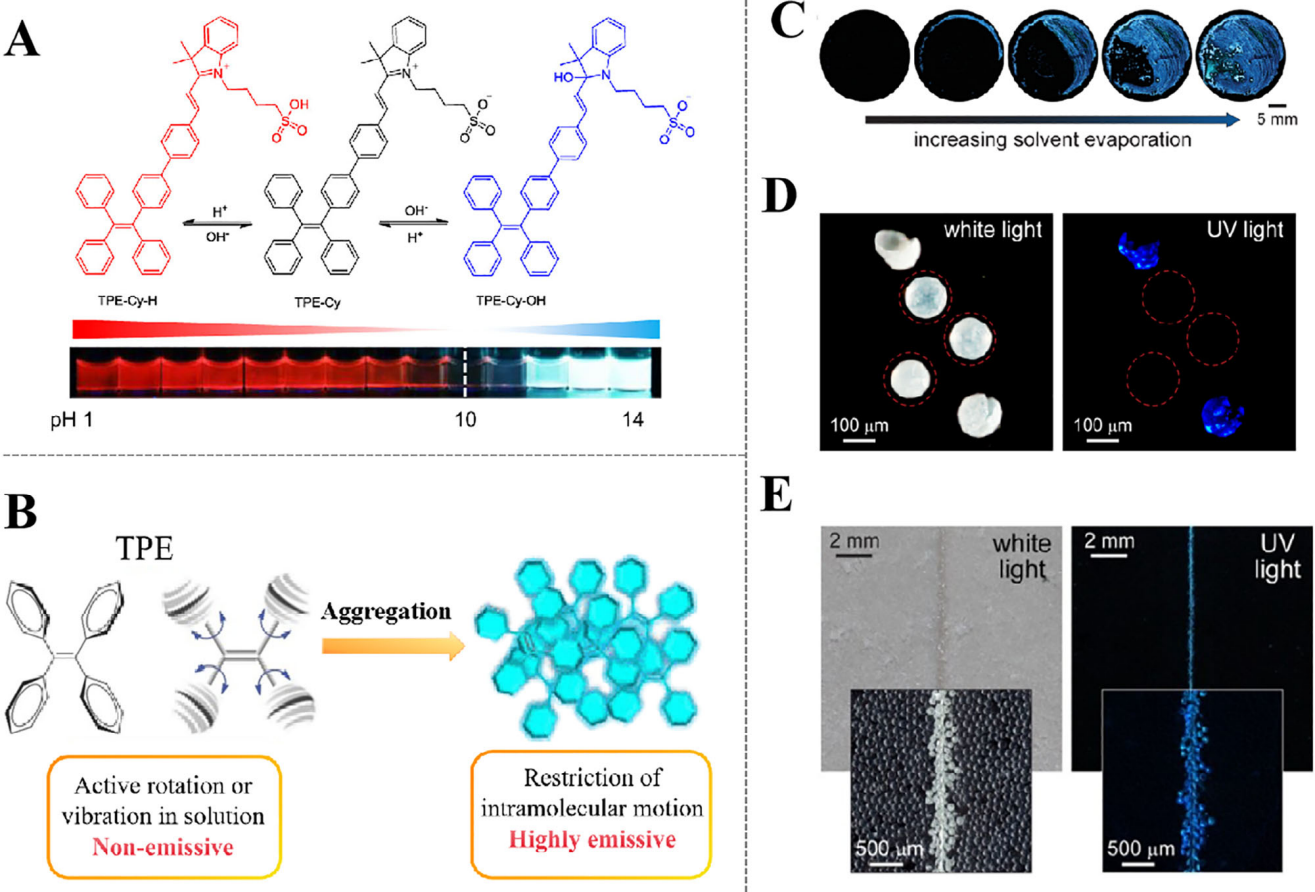
The characteristics of Aggregation‐induced emission (AIE) indicators. (A) working principle: fluorescent response of TPE‐Cy to pH changes. Reproduced with permission [76]. Copyright 2013, American Chemical Society. (B) AIE mechanisms with the examples of TPE. Reproduced with permission [75]. Copyright 2014, Royal Society of Chemistry. (C) photographs of a TPE solution under illumination with UV light. Reproduced with permission [78]. Copyright 2016, American Chemical Society. (D) stereomicrographs of TPE microcapsules under illumination with white light and UV light. Reproduced with permission [78]. Copyright 2016, American Chemical Society. (E) photographs of an epoxy coating containing 10 wt% TPE microcapsules under illumination with white light and UV light after being scratched with a razor blade. Reproduced with permission [78]. Copyright 2016, American Chemical Society.

AIE luminogens (AIEgens) have widespread applications on biology, sensing, and optoelectronics industry [72, 73]. In solution condition, AIEgens intramolecular rotation/vibration absorb photon energy in a non‐radiative chiral manner, resulting in non‐emission of AIEgens [66, 74]. When AIEgens are aggregated into solid state, the intramolecular motion is constrained so that fluorescence signals are dramatically enhanced. Luminescent molecules with AIE properties are routinely used as fluorescent indicators, which create fluorescent signals of sufficient brilliance and effectively obviate the behavior of aggregation‐induced fluorescence being “turned off” [75]. A common coating strategy is to encapsulate AIE molecules in containers, once the coating is damaged and destroys microcapsules, resulting in rapid aggregation of AIEgens and fluorescence signals strengthen after solvent evaporation. Recently, a pH‐responsive agent consisting of tetraphenylethene (TPE) and cyanine (Cy) units, which can react with OH^−^/H^+^ to sense pH flux in a broad‐based and the broadest range to date (Figure 8A) [76]. Furthermore, the information reflect by the fluorescence signals of AIE‐based coatings represents coatings damages rather than present metal corrosion, and implicate that the AIE‐based coatings may act early [77].

**FIGURE 9:**
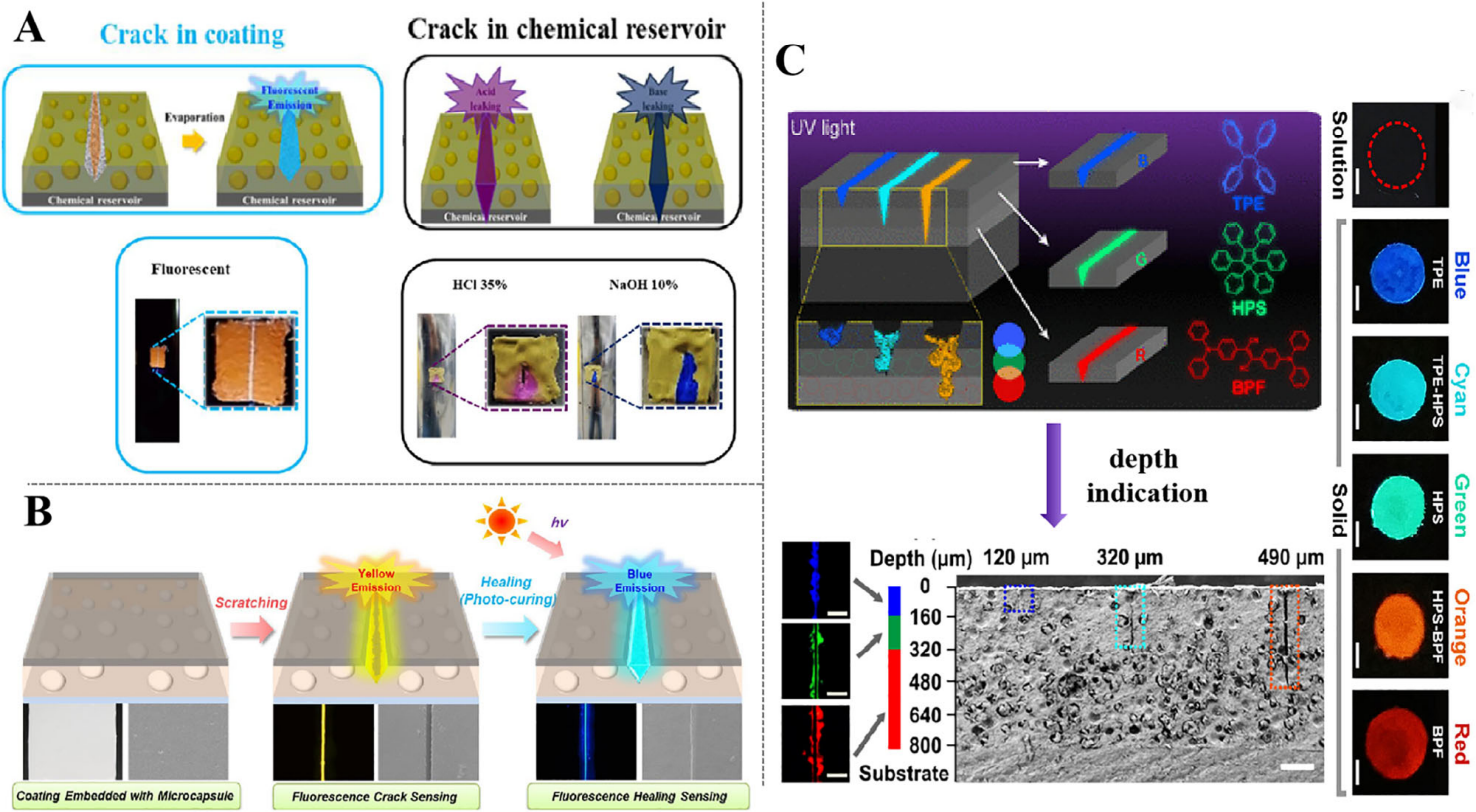
Utilization of AIE indicators in self‐warning coatings. (A) the DSRTET coatings to the laboratory scale chemical reservoirs: actual crack test. Reproduced with permission [46]. Copyright 2018, Elsevier. (B) schematic diagram of the fluorescence system used to detect cracks with yellow emitted light and detect healing with blue emitted light. Reproduced with permission [80]. Copyright 2018, Elsevier. (C) schematic of the damage indication in multilayer coatings with varying cracks depth and molecular structures of BPF, HPS, and TPE, as well as typical color blending among red, green, and blue are included. Reproduced with permission [47]. Copyright 2018, American Chemical Society.

Tetraphenyl ethylene (TPE) is a typical organic fluorescent molecule with AIE behavior and also a promising mechanically triggered indicator in self‐warning coatings. In aggregated state, TPE molecules can't pass through π─π stacking process due to the shape of propeller, while the intramolecular rotation of its aryl rotor is highly restricted under physical limitations (Figure 8B) [79]. It can be observed in Figure 8C, the solution did not illuminate under UV irradiation, but a luminous blue fluorescence was perceived in solid residue formed by solvent evaporation. Intact and ruptured microcapsules had completely divergent fluorescence performance under two light sources (Figure 8D), demonstrated that the potential application of TPE microcapsules in self‐warning coatings. 10 wt% TPE microcapsules were added to transparent epoxy coating and the photographs of scratched coating under white and UV lights were shown in Figure 8E, the significant enhancement in visual identification of damaged areas by UV light. The coating also had a precise detection down to cracks of 2 µm in size, which was important for timely repairing [78]. TPE fluorescent molecules are also used in factories to detect acid or alkali environment. The experimental results from Lee et al. [46] appeared that TPE microcapsules in thiol‐epoxy thermosetting coatings (DSRTETs) containing thymol blue (pH indicator) for cracks detection, and different fluorescence can be showcased in response to pH changes. As shown in Figure 9A by actual cracks testing that the color of DSRTET coating changed from light green to red in acidic solution and become blue in basic solution. This dual stimulus responsive coating is very useful for detecting both cracks in coating materials and chemical leaks caused by burst tanks.

Further studies, AIE‐based coatings can not only help people identify coating damages, but also detect the degree of self‐repairing concurrently. Song et al. have carried out a lot of works [80, 81, 82], as shown in Figure 9B, yellow fluorescence had warning of cracked region and blue fluorescence was emitted to indicate self‐repairing regions after microcapsules released repair agents. Self‐repairing coatings encapsulated with 25% microcapsules balanced the sensitive fluorescence detection and the superior repairing efficiency of damage zones [80]. The detection and estimation of small‐scale damages at early stages are essential for coatings to extend their lifetime, which can avoid further disastrous structural failure. Multiple reagents integrated by Lu et al. [47] who proposed an approach to warn of damages in multilayered polymer coatings by blending three color‐specific AIEgens with coating substrates. Three capsules of TPE (blue), HPS (green), and BPF (red) were used embedded in the top (thickness 160 µm), middle (thickness 160 µm), and bottom (thickness 480 µm) layers of the coating. Due to unequal depth of scratches, the coating layers were penetrated, different combinations of AIEgens were activated to visualize detect the depth of damages based on corresponding fluorescent colors. In this way, the extent of coating damages could be visually assessed by observing counterpart color as shown in Figure 9C, which facilitated the prompt repairing of synthetic manner. The utilization of AIEgens in self‐warning coatings provides a direct and effective method for rapid detection of early microcracks and delivers a versatile detection strategy compatible with different manufacturing processes.

#### 3.3.2 Visible Coloration of CVL for Damage Detection

**FIGURE 10:**
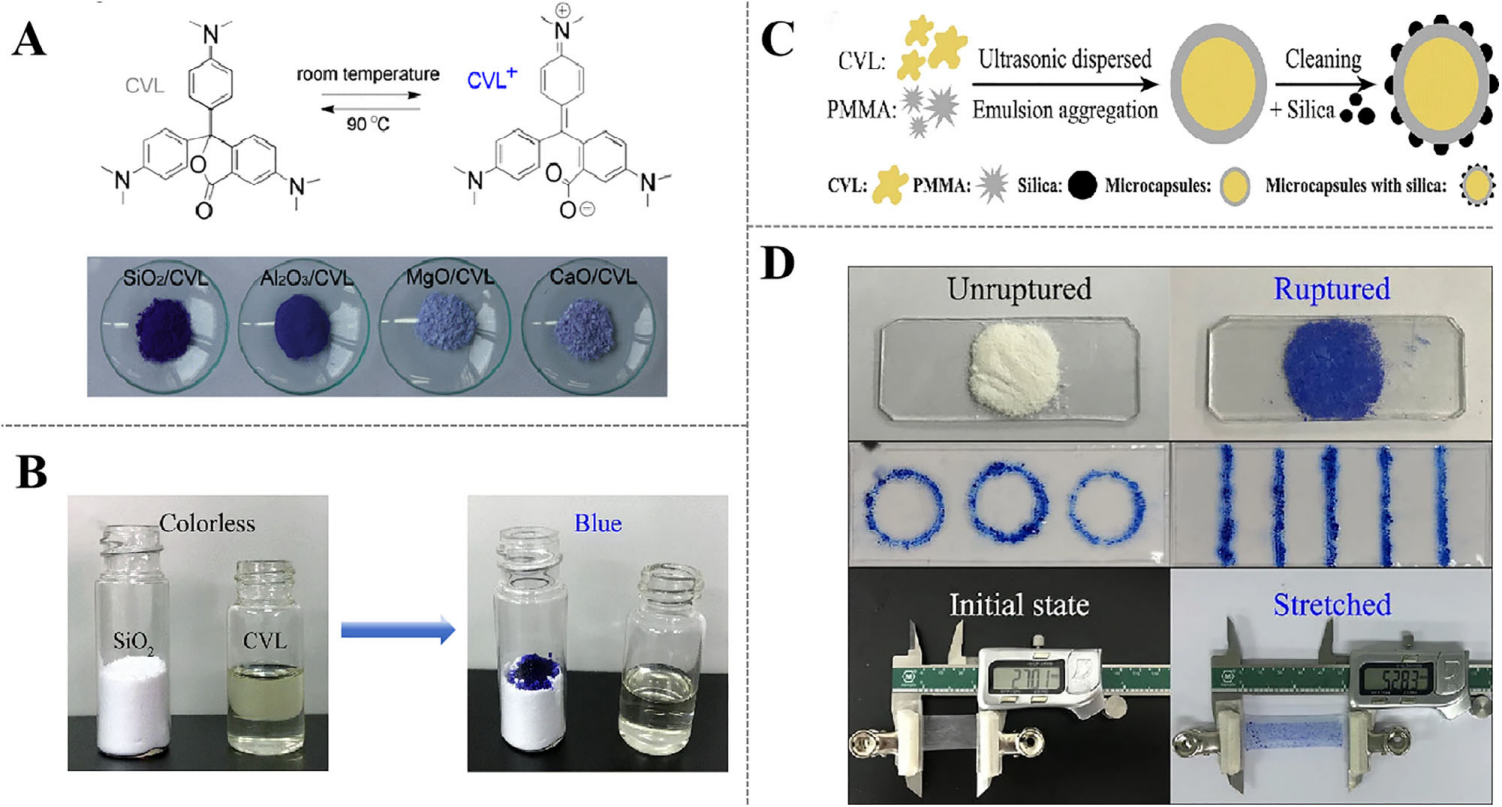
Applications of CVL indicator in self‐warning coatings. (A) Temperature‐induced reversible switching between CVL and CVL^+^ (top) and images of SiO_2_/CVL, Al_2_O_3_/CVL, MgO/CVL, and CaO/CVL (bottom). Reproduced with permission [83]. Copyright 2016, American Chemical Society. (B) Color development reactions of the CVL dye. Reproduced with permission [85]. Copyright 2020, Springer. (C) A schematic diagram of the fabrication process of SiO_2_/PMMA/CVL visual microcapsules. Reproduced with permission [85]. Copyright 2020, Springer. (D) Self‐reactive microcapsule as visual sensor for damage reporting. Reproduced with permission [85]. Copyright 2020, Springer.

CVL is favored as mechanically triggered indicator for coatings owing to its selective reactivity with a broad spectrum of oxides via hydrogen bonding, which can show drastic and speedy changes in color rendering. The lactone ring of CVL undergoes ring‐opening in the presence of weak acids or proton donors (dilute HNO_3_ solution and SiO_2_), leading to the formation of triphenylmethane (CVL^+^) with an intense blue color. When encountering hydroxyl containing oxides (e.g., SiO_2_, Al_2_O_3_, CaO, and MgO), the order of color depth of hybrids is observed: SiO_2_/CVL > Al_2_O_3_/CVL ≫ MgO/CVL ≈ CaO/CVL, which is consistent with the order of hydrogen bonding donating capability of the oxides (Figure 10A) [83]. As shown in Figure 10B, CVL solution is dropped into SiO_2_ powder and then dark blue color appeared rapidly, proving that two substances have great potential for visual self‐warning feature. Wang et al. [84] encapsulated CVL into polymethylmethacrylate (PMMA) by ethyl acetate solution of CVL as an indicator, the microcapsules were embedded in polymer coating. When cracks propagate in the coating, CVL in leuco form was released from ruptured microcapsules and reacted with silicon dioxide in concrete, which can highlighted damaged areas by remarkable blue color. There was also a study on mechanical properties of microcapsules, which designed self‐reactive visual microcapsules via solvent evaporation method (Figure 10C). Microcapsules under the force of shear, pressure and tension, white microcapsule powder were crushed and turned into intense blue (Figure 10D) [85]. Therefore, self‐reactive visualized microcapsules have great application prospects in self‐warning of mechanical abrasion, pressure and tensile damages on coating surface.

Besides, we also need to consider other factors on the microencapsulation technology of mechanically triggered indicators, especially the encapsulation of CVL solution has a great test for barrier and mechanical properties of microcapsule shell. The solvent in capsule core should be retained for a long enough period of time so that not to be contaminated by external environment. To further improve the stability of microcapsules, other techniques can be applied, such as incorporation of double‐layer microcapsules or protective surface coatings. Double‐layer microcapsules with good solid‐stability at 180°C were prepared by using PDA polymer [48]. The extraordinary thermal and solvent stability of PDA‐coated microcapsules make them promising candidates for self‐warning polymers and composites cured at elevated temperatures, as well as for applications in a variety of fields where capsule stability is critical.

Self‐warning coatings can be applied to oil and gas pipelines and storage tanks to monitor corrosion and damage, ensuring the integrity and safety of oil and gas facilities. This monitoring mechanism can promptly alert operators to potential leaks or failures, reducing production interruptions and environmental hazards. Currently, most self‐warning coatings are developed based on transparent coatings, but commercial coatings often include fillers that render them opaque. Insufficient light transmission can easily lead to false alarms or missed signals. To meet market needs, further research is required to develop early self‐warning coatings suitable for opaque or colored coatings.

## 4 How to Achieve Self‐Repairing Performance

Motivated by biology, self‐repairing coatings are designed to address structural or functional degradation caused by external actions, which can restore certainly degree of protective performance, thus realizing prolonged metal protection. The mechanisms are predominantly categorized into intrinsic self‐repairing and extrinsic self‐repairing [86, 87]. Intrinsic self‐repairing coatings utilize dynamic reversible chemical bonds or physical interactions within the polymer matrix to recover coating properties. Hence, this restorative property is fundamentally liberated from the limitation of metal substrates. Conversely, extrinsic self‐repairing coatings are characterized by the incorporation of responsive micro/nano containers loaded with corrosion inhibitors or repair agents [88, 89, 90]. Microcapsules crack under mechanical stress, and releasing healing agents that polymerize to form protective films in coating defects. Self‐repairing coatings embedding responsive micro/nano containers, which can be discharged from containers under corrosive environment stimulus, such as pH variations, redox potential, corrosive ions, light, magnetic field temperature, etc. In principle, extrinsic self‐repairing coatings typically involve interactions with the indigenous environment of damages or corrosion areas.

When evaluating the potential applications of self‐repairing organic coatings, it is essential to mention the automotive industry, where various commercial products are already available. Most of these products are ceramic coatings primarily composed of inorganic materials like silicon dioxide rather than polymer binders. These upmarket automotive coatings can repair small, visible cracks, such as key scratches, either through heating or by exposure to sunlight over several days. Additionally, some commercial self‐repairing coatings incorporate shape memory polymers into ceramic systems. These polymers deform when subjected to mechanical damage, like scratches, and then revert to their original shape upon heating, effectively repairing the coating.

### 4.1 The Mechanisms of Intrinsic Self‐Repairing Coatings

Intrinsic self‐repairing coatings are repaired by restoring the inherent bonding of the polymer networks in coating matrix by chemical interactions or physical interactions. Chemical interactions generally encompass reversible covalent bonding, whereas physical interactions are mostly related to reversible non‐covalent bonding. The thermoreversible bonds can disintegrate when heated up to a certain temperature, allowing the polymer chains to flow to the defects and re‐crosslink to repair the defect [27, 91]. The primary advantage of such self‐repairing systems lies in their capacity for potentially limitless repair cycles, obviating the need for secondary healing agents. Given the reversibility of covalent and non‐covalent bonds, and the absence of a depletable healing agent, intrinsic repairing process can be repeated a theoretically infinite number of times in the same place. Additionally, intrinsic self‐repairing does not require microcapsules or other larger structures within the polymer matrix. This implies that the performance of many coatings, particularly optical and tensile strength, are likely to remain unaffected. However, external stimulation from non‐corrosive courses are pivotal as they supply the requisite activation energy for bond disruption and reformation. Intrinsic self‐repairing coatings are categorized here into chemical and physical interactions for the purpose of elaboration. Table 2 lists the types of different interactions in intrinsic self‐repairing coatings.

**TABLE 2:** Types of different interactions in intrinsic self‐repairing coatings.

Type	Coatings	Bonding method	Repairing efficiency	Reference No
Chemical interactions	Aniline trimer derivative	Diels‐Alder	99.2%	[92]
	Polyurethane acrylate	Diels‐Alder		[93]
	Epoxy resin coating	Disulfide bonds	93.68%	[94]
	Polyurea‐urethane/epoxy blend	Disulfide bonds	80%	[95]
	Waterborne polymer	Imine bonds		[96]
	Polyurethanes	Cinnamoyl	100%	[97]
	Epoxy‐based network polymers	Coumarin	90%	[98]
	Epoxy polymers	Anthracene	99%	[99]
Physical interactions	Polyurethane	Hydrogen bonds		[100]
	Polydimethylsiloxane	Hydrogen bonds	85%	[101]
	Polyurethane oligomers	Hydrogen bonds		[102]
	Epoxy coating	Hydrogen bonds	99.7%	[103]
Mixed interactions	Polyurethane oligomers	Hydrogen bonds and Diels‐Alder	100%	[104]
	Epoxy‐modified polyurea	Hydrogen bonds and Diels‐Alder	89%	[105]
	Waterborne polyurethane	Hydrogen bonds and ionic bonds	92.43%	[106]
	Polydimethylsiloxane	Hydrogen bonds and disulfide bonds	94.06%	[107]
	Polyurethane prepolymers	Imine bonds and disulfide bonds		[108]
	AgNP hybrid silicone coating	Disulfide bonds, oxime urethane bonds, metal coordination bonds, and hydrogen bonds	91.7%	[109]

#### 4.1.1 Self‐Repairing Based on Chemical Interactions

**FIGURE 11:**
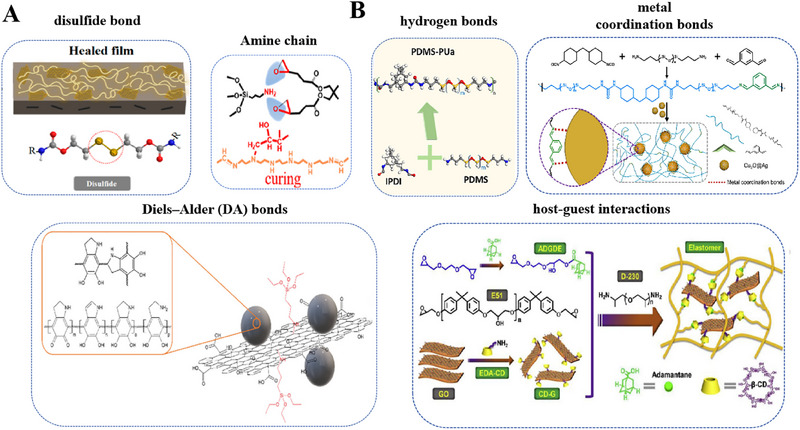
Types of (A) reversible covalent bonds and (B) reversible non‐covalent bonds. Adapted with permission.[110] Copyright 2021, Elsevier. Adapted with permission [111]. Copyright 2019, Elsevier. Adapted with permission [112]. Copyright 2022, Elsevier. Adapted with permission [113]. Copyright 2021, Elsevier. Adapted with permission [114]. Copyright 2021, MDPI. Adapted with permission [115]. Copyright 2021, Elsevier.

Upon exposure to light or heat stimuli, the flow properties of polymers are optimized, which can strengthen their reaction by tightening the broken bonds. Optics and thermodynamics entail the interaction between optical and thermal energy signals, while the interplay between bonds plays a crucial role in various optical and thermal applications, enabling precise control and manipulation of the optical and thermal properties of coatings [116, 117, 118]. Especially UV light, which can provide the activation energy required for bond breakage/reformation, this conclusion was first substantiated in 1947 [91]. An early work demonstrated that disulfide bonds can undergo exchange reactions between different molecules to form new compounds under UV light (312–577 nm) irradiation from 400 w high‐pressure mercury lamp [119]. This conclusion implied that disulfide bonds can recover through both heat and light exchange reactions. Several reversible covalent bonds as chemical interactions have been incorporated in photothermal self‐repairing coatings, including Diels–Alder (DA) bonds [92, 93, 105], disulfide bonds [114, 120] imine bonds [110], and transesterification (Figure 11A) [121].

**FIGURE 12:**
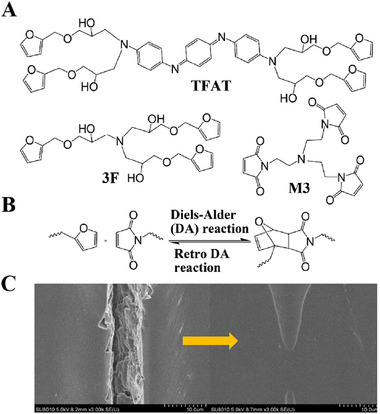
Harnessing chemical interactions for self‐repairing mechanisms. (A) chemical structures of the compounds used in building up the self‐repairing anti‐corrosion coating materials. (B) thermally‐reversible Diels‐Alder reaction between furan and maleimide groups. (C) SEM image of the coating showing thermally activated self‐repairing behavior. Reproduced with permission [92]. Copyright 2017, Elsevier.

A typical example is the use of Diels–Alder (DA) reaction, that is a [4 + 2] cycloaddition between conjugated diene and dienophile, including well‐studied pair of furan and maleimide [122]. In one case, Wei et al. [92] prepared an aniline trimer derivative possessing multiple furan groups (TFAT) and used them as building units for assembling self‐repairing coatings (Figure 12A). Based on corrosion current density calculations, the protective properties of scratch coatings were fully restored after heating at 140°C for 1 h because induce the DA inverse reaction, and then re‐crosslinking reactions were accomplished by DA reaction at 80°C for 24 h. When the coating were mechanically damaged by external force, the DA reverse reaction occurred after heat treatment with break chemical bonds (Figure 12B). During slow cooling, the DA reaction occurs due to molecular chain movement and ionic bonding, and polymer is re‐crosslinked to achieve self‐repair of the coating (Figure 12C). In order to make networks distribution more homogeneous than free radical polymerization, fresh idea that used two thiol monomers as raw materials and compounded thiol‐alkenes with DA bond‐containing urethane resins. Novel UV‐curing urethane coatings exhibit excellent self‐repairing properties at lower temperatures (90°C) and shorter times (35 s), even the coatings realize self‐repairing function at lower temperature have wider practical application prospects [93].

Other photodynamic dynamic chemical groups such as cinnamoyl, coumarin, anthracene, which are subjected to reversible [2+2] or [4+4] photodimerization and photocleavage reactions in the presence of UV light. When microcracks occur in coatings, free photosensitive groups under UV irradiation will be crosslinked again, thus performing self‐repairing assignments autonomously. Yan et al. [97] brought cinnamoyl structure into polyurethane chains as side group to produce a new type of dynamically photocrosslinked polyurethane. Crosslinking occurred under 300 nm UV irradiation, and photocrosslinking led to chain expansion followed by crosslinking of the polyurethane. The polymer repairing is driven by subsequent uncrosslinking reaction under shorter wavelength radiation, and the addition reaction leads to the recovery of double bonds. Coumarin, which is structurally similar to cinnamoyl derivatives. Two photoreversible 4‐armed coumarin monomers (4ACMs) by epoxidation of coumarin and diamine chains reacted with different chain lengths, and 4ACMs underwent reversible dimerization under UV light. The polymers formed not only have remarkable self‐repairing ability, but also have terrific mechanical strength [98]. The photodimerization reaction of anthracene and its derivatives is similarly reversible, and free anthracene can be regained under UV irradiation at a certain wavelength or thermal annealing. Later, further studies that used two anthracene‐based diamine cross‐linking agents to obtain four highly photoreversible crosslinked epoxy polymer coatings, and self‐repair properties were achieved by photoreversible cleavage of the anthracene dimer [99].

Photosensitizing groups are introduced into the main or side chains of polymers, making them promising for applications not only in the field of self‐repairing coatings, but also in smart materials, such as electrochemical sensors and devices, and electronic skin [123]. The fluidity of coating matrix allows a large amount of materials with reversible chemical bonds to be transferred to damaged area in self‐repairing coatings. However, this most often requires a great deal of motivational energy from the external environment.

#### 4.1.2 Self‐Repairing Based on Physical Interactions

Except for the actions of reversible covalent bonds to effect on intrinsic self‐repairing, non‐reversible covalent bonds known as physical actions are also more practical means for self‐repairing, such as hydrogen bonds [100, 105, 124], metal‐ligand [115, 125], ionic bonds [106], and host‐guest interactions (Figure 11B) [126, 127]. Hydrogen bonding is a quintessential type of reversible non‐covalent bond, which exhibits slightly stronger interaction than intermolecular force (van der Waals force) but is weaker than covalent and ionic bonds [128, 129].

**FIGURE 13:**
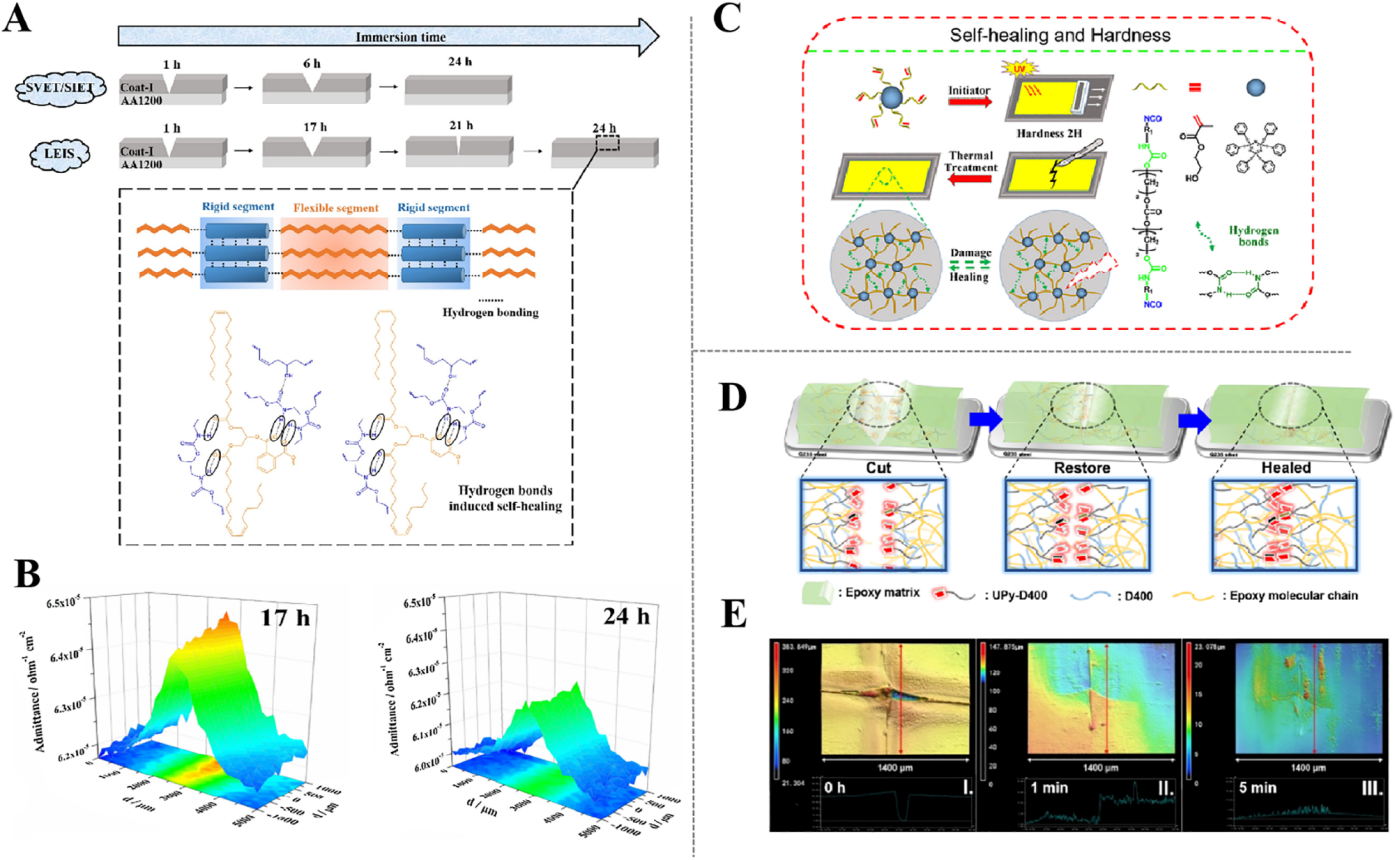
Harnessing hydrogen bonds for self‐repairing mechanisms. (A) Illustration of the self‐repairing process of Coat‐I in NaCl solution. Reproduced with permission. (B) Admittance evolution over the coated sample Coat‐I containing an artificial defect [100]. Copyright 2020, Elsevier. (C) Schematic diagram of preparing high hardness self‐repairing coatings using molecules with a hard core and soft arm structure of UPy dimers. Reproduced with permission [102]. Copyright 2018, American Chemical Society. (D) The self‐repairing process of the UPy‐epoxy coating. (E) The CLSM images of the recovery process of UEP‐3 material on cross‐shaped damage. Reproduced with permission [103]. Copyright 2021, Elsevier.

Generally, the polyurethane structure consists of two types of segments, rigid and flexible parts [130, 131]. The flexible segments can be synthesized from vegetable oils and organic chain extensors by transesterification reaction [132]. This hypothesis inspired Nardeli to develop self‐repairing polyurethane coating based on hydrogen bonding by adjusting flexible ratios and rigid chain segments. Due to the higher number of flexible chain segments in Coat‐I (3:2), these bendable segments (polyesters) contribute to the formation of more hydrogen bonds and highly developed cross‐linking structure, which given Coat‐I superior self‐repairing effect (Figure 13A). Figure 13B shows the LEIS maps of Coat‐I during immersion for 17 h and 24 h. Its value decreased over time, indicating that the substrate became protected, probably, due to coating recovery [100]. Liu et al. [102] synthesized “hard core, flexible arm” polyurethane (PU) with photo‐sensitive groups by using rigid group as “core” and flexible segment as “arm”. In addition, the UV‐cured self‐repairing PU coatings were prepared by designing some coating formulations with PU oligomers. As shown in Figure 13C, the urethane structures in flexible chain segments form hydrogen bonds between molecular chains, enabling the coating to undergo network reconfiguration under heating condition.

Since reported by Meijer [133], 2‐ureido‐4‐pyrimidinone (UPy) has been used as robust, selective, and directed hydrogen bonding system in various supramolecular polymers and reversible networks. UPy can form supramolecular dynamic interactions through dimerization with highly oriented self‐complementary donor–donor‐acceptor–acceptor hydrogen bonds [134]. For example, UPy motifs are grafted onto flexible cross‐linker polypropylene glycol bis(2‐aminopropyl ether) (D400), which is a reliable method to introduce hydrogen bonding into epoxy networks [103]. An intrinsic self‐repairing coating based on UPy quadruple reversible hydrogen bonding was prepared by using Upy‐D400 epoxy chains (Figure 13D). Both higher mobility of the epoxy chains at room temperature and high self‐bonding constant (>106 m ^−1^) of UPy bonds accelerated progress on self‐pairing of the coating, the CLSM images displayed in Figure 13E show that the coating could be fully repaired within 5 min even from a severe cross‐cut. The self‐repairing ability is mainly depended on cross‐linking degree of the coating, which is enhanced by increasing double bond contents. However, higher cross‐linking degree will hinder the free movement of polymer chains in the cracks, so that hindering the remodeling of hydrogen bonds.

**FIGURE 14:**
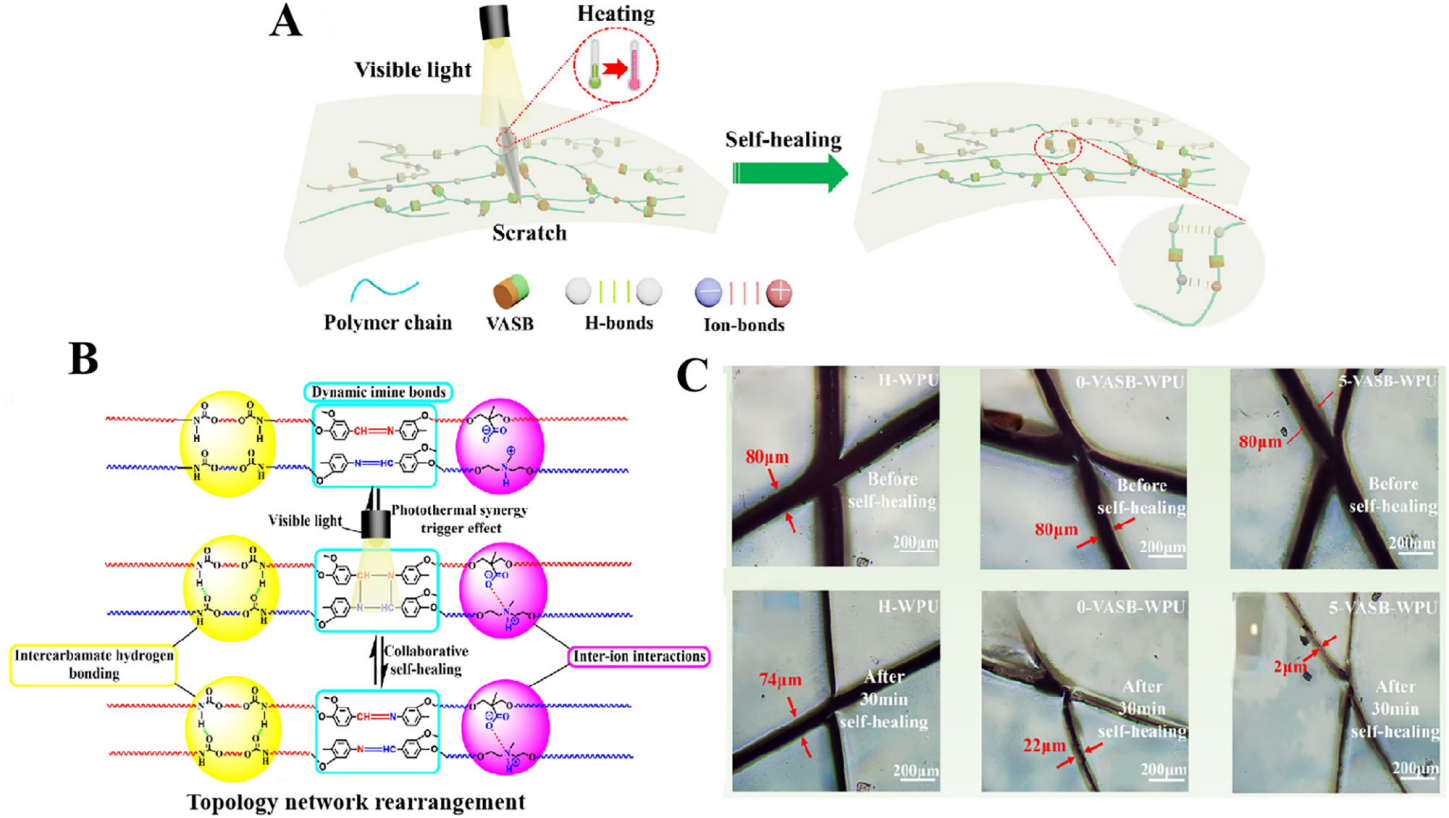
(A) Schematic of the self‐healing process in the VASB‐WPU topology network under the condition of photo‐thermal co‐triggering. (B) Schematic of the synergistic interaction of dynamic chemical bonds in VASB‐WPU. (C) Optical microscopic images of H‐WPU, 0‐VASB‐WPU, and 5‐VASB‐WPU before healing and after self‐healing for 30 min at 300 mW/cm^2^. Reproduced with permission [106]. Copyright 2023, Elsevier.

The use of dynamic covalent bonding strategies can bring efficient repair properties, the low activation energy (Ea) for bond exchange cannot maintain the basic mechanical properties of the material. The strength of the supramolecular action is not enough to form a stable repair structure at the breakage. A key challenge lies in achieving a balance between rapid self‐repairing properties and high mechanical strength, where the synergistic effect of multiple repair mechanisms results in materials with different shape memory properties and multiple repair effects [113, 135]. For instance, it is reported that the self‐repairing coatings contained a double cross‐linked network system based on hydrogen bonds and D–A reaction [104]. With the addition of DA monomer, the repair efficiency of the coating was greatly improved, and even damaged coatings could be completely repaired within 60 s. Chen et al. [106] synthesized a vanillin‐like aromatic Schiff base (VASB), which is responsive to both light and heat (Figure 14A). VASB was used as a chain extender to introduce the imine bond into the main chain of a waterborne polyurethane (VASB‐WPU) resulting from the introduction of physical interactions (hydrogen and ionic bonding) in the side chains (Figure 14B). By implementing a healing process that involved light and heat stimulation, the VASB‐WPU exhibited an impressive healing efficiency of 92.43% in thin films while maintaining exceptional mechanical performance (Figure 14C), with a tensile stress of 10.59 MPa and a toughness of 16.56 MJ/m^3^.

### 4.2 The Mechanisms of Extrinsic Self‐Repairing Coatings

Extrinsic self‐repairing coatings are characterized by the incorporation of encapsulated reagents with inhibitory efficacy that are embodied in the host coatings and await the controlled release of environmental stimuli, which is critically rely on the selection of containers and restorative agents. Currently, the controlled release of containers can be summarized in two primary pathways: one involves the immediate response to mechanical damages, leading to the outflow of corrosion inhibitors, the other leverages the stimulation of micro‐environment associated with the corrosion process or external energy sources to trigger the containers, facilitating the release of corrosion inhibitors.

#### 4.2.1 Mechanically‐Triggered of Microencapsulated Self‐Repairing Coatings

**FIGURE 15:**
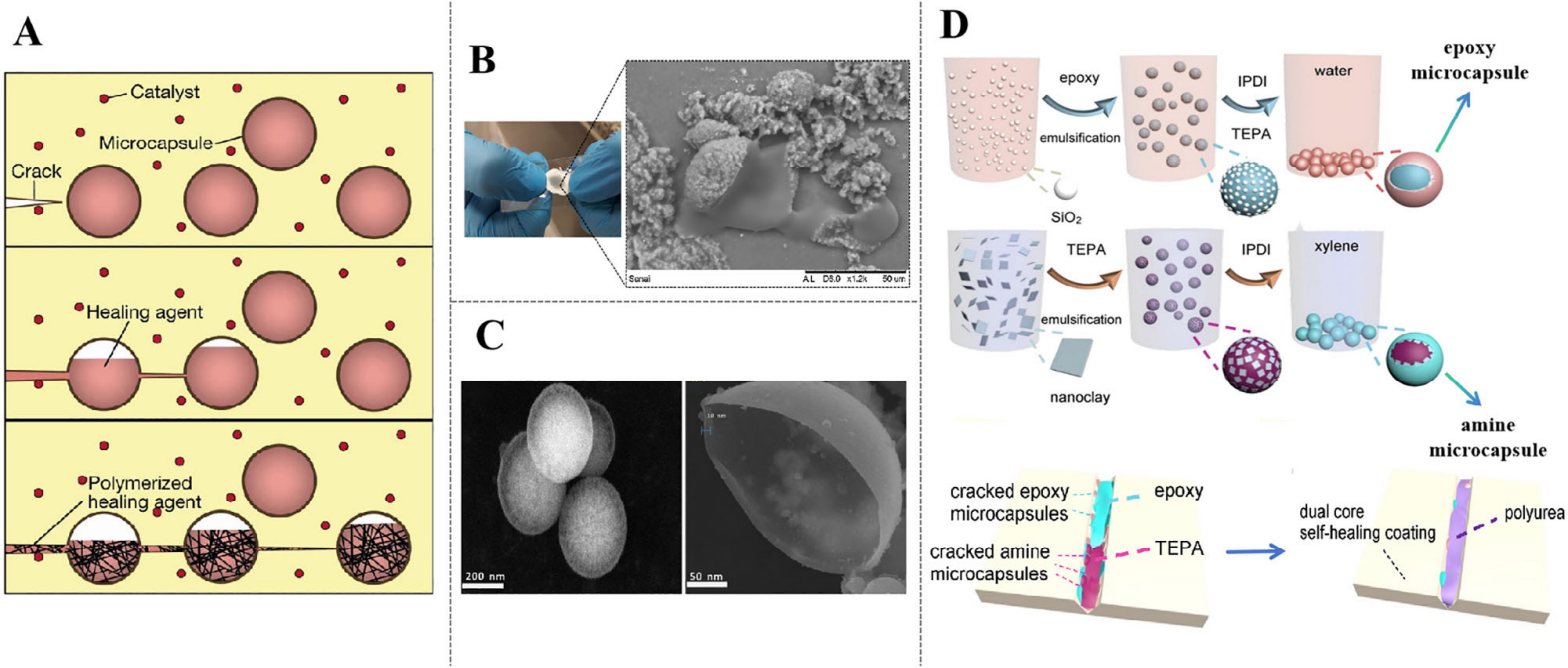
Micro‐encapsulated self‐repairing coating systems. (A) A microencapsulated healing agent is embedded in a structural composite matrix containing a catalyst capable of polymerizing the healing agent. Reproduced with permission [144]. Copyright 2001, Macmillan Magazines Ltd. (B) Image of the release test of the microcapsule core material by mechanical pressure. Reproduced with permission [151]. Copyright 2020, Elsevier. (C) SEM images of UF microcapsules: active agent loaded (left) and active agent leached out (right). Reproduced with permission [152]. Copyright 2015, Elsevier. (D) Synthesis process of epoxy microcapsules, amine microcapsules and the coating repair process. Reproduced with permission [153]. Copyright 2021, Elsevier.

Microcapsules are more desirable options as mechanical stimulus response containers, based on the fact that polymers with decent film‐forming nature and mechanical performance undergo chain‐to‐chain breakage or entanglement under mechanical forces, which can lead to microcapsules rupture [136, 137]. Currently, polymers are used as wall materials of micro‐capsules including melamine resins [138], urea‐formaldehyde resins [139], polyurethanes [140], polystyrene [141], and polyaniline [142, 143]. In first generation of self‐repairing system, a classic example was reported by White and Sottos [144], the research practiced that encapsulated liquid repairing agents and endo‐dicyclopentadiene (endo‐DCPD) to shape several granular micro‐spheres, then solid phase Grubbs'catalyst were embedded in epoxy coating matrix separately. When cracks cut through inside the films, microcapsules were ruptured and released repairing agents into damage regions, where the polymerization upon mixing with the catalyst phase (Figure 15A). It is a dual‐component material that fulfills the self‐repairing role, which is very advantageous for the overall performances of coatings while simplifying the additional materials.

Lately, healing agents that fulfill repair behaviors individually are taking over microcapsule self‐repairing coatings, including drying oils [145, 146, 147], epoxy resins [148], organosilanes [149], and aliphatic amines [150]. As the most predominant feature, their restoration process don't require any catalyst and only need to come into contact with atmospheric oxygen to form salvage layer. Microcapsules must release fluid repair materials when they are sabotaged by mechanical pressure, it means that capsules need to be dispersed uniformly in coatings to prevent existing monitor blind spots. To resolve issues like these, some attempts at the microencapsulation process were gotten tests, by using the in situ polymerization method to prepare UF microcapsules containing Tung oil, focused on spherical, mononuclear, and appropriately sized microcapsules [151]. The stimulation reactivity of microcapsules was demonstrated in Figure 15B, which visualized rapid release of additives under mechanical force. They also proposed a possible stimulus‐responsive mechanism caused by the blistering phenomenon that appeared in all specimens, even so, it was justified that the presence of functionalized microcapsules can delay corrosive process. In order to improve corrosion resistance of the coatings, an option that add one more corrosion inhibitors to microcapsules. Siva et al. [152] prepared microcapsules loaded with both linseed oil and inhibitor (MBT). It can be observed that the inner layer of microcapsule shell was smooth and void‐free, the outer surface was rough, with the thickness of 10 nm (Figure 15C). Once microcapsules were ruptured, the inhibitor and linseed oil were synchronously released to form an insoluble compound, as well as linseed oil formed an intact layer after oxidized in the air. Compared to the microcapsule coating system with only linseed oil, the dual‐component coating system showed better corrosion resistance.

Among microcapsule‐based self‐repairing coating examples, epoxide amine systems are considered as one of the highly efficient execution strategies. Self‐repairing characteristics of epoxy amine microcapsules are attributed to the release of epoxy linkages from amine/amide or self‐polymerization of epoxy groups under alkaline conditions [153]. As shown in Figure 15D, polyurea (PU) shell microcapsules loaded with epoxy and amine were synthesized by interfacial polymerization of diisocyanate and amine based on O/W and W/O Pickering emulsion templates respectively. Upon coatings were mechanically damaged, microcapsules were ruptured and self‐polymers formed by the chemical reaction of epoxy resin, after TEPA flowed out of core to repair scratched areas. Lee group encapsulated an epoxy (diglycidyl ether of bisphenol A) and a hardener (mercaptan/tertiary amine) within an alginate biopolymer to form self‐repairing multicore. The dual‐capsule self‐repairing system showed three to four repairing cycles, whereas the capsule‐catalyst self‐repairing system only demonstrated two to three repairing cycles [154]. It is no doubt that the loading rate of microcapsules and the activation period of repair agents are influential for self‐repairing coatings to achieve multiple and sustainable self‐repairing.

Obviously, the modal of generic microencapsulated coatings can only withstands the first self‐repair. If there is secondary destruction, it can't contribute to repair once again. This is due to the limitation amount of microcapsules and the curing feature of functional pharmaceutical itself. Yang, for example, developed an organogel‐based self‐repairing coating with strong resistance to secondary damages in repaired regions, due to the repair agents (organogel precursor material) filled into damaged areas would form viscoelastic organogel. No secondary damage was observed after the repaired coating was subjected to severe vibration. Organogel‐based self‐repairing coatings prevent secondary deterioration because of the viscoelasticity of the organogel [155]. However, which is more important, the restoration of barrier properties or the restoration of mechanical strength, it is a question worth thinking about. The experimental attempts by Lee et al. [156] showed that viscoelastic substances were used as repairing agents, unlike the former, they focused on the ability to maintain repairing state when the secondary cracks extended. By conducting water sorptivity test at each crack width, they also evaluated the self‐repairing efficiency of the coating system and its ability to remain in a repaired state. It appeared that highly repairing efficiency of 90% at a 150 µm crack width and attained repairing efficiency of about 80% up to the crack width of 350 µm. This research objectively illustrated that focus on secondary cracks is a promising system for regenerative coating, which can undergo cracks formation and expansion.

The satisfactory self‐repairing performance of the coatings largely rely on the size, quantity and distribution of microcapsules, as well as their compatibility with coating matrix. Ideally, the walls of the microcapsules should be hard enough to ensure complete separation of agents and mechanical properties of coatings, especially most of reactive repairing agents are liquids. To date, microcapsules are typically prepared between tens and hundreds of micrometers in diameter [157]. However, for thin anti‐corrosion coatings (particularly less than 100 µm) [138, 158], they can't be totally concealed by coatings.

#### 4.2.2 Stimulus‐Responsive Mechanism of Self‐Repairing Coatings

It can be seen from above precedents that most of the microcapsules are responsive to mechanical damages. Nevertheless, the types of stimulus response and applications scope of such nanocontainers are relatively modest, accordingly, self‐repairing coatings also can control the release of their encapsulated contents under various environment stimulation. Currently, after surface modification of micro/nano containers can be designed to achieve controlled release of corrosion inhibitors by effectively respond to internal or external microenvironments, including pH, redox, corrosive ions, light, magnetic‐fields [159, 160].

**FIGURE 16:**
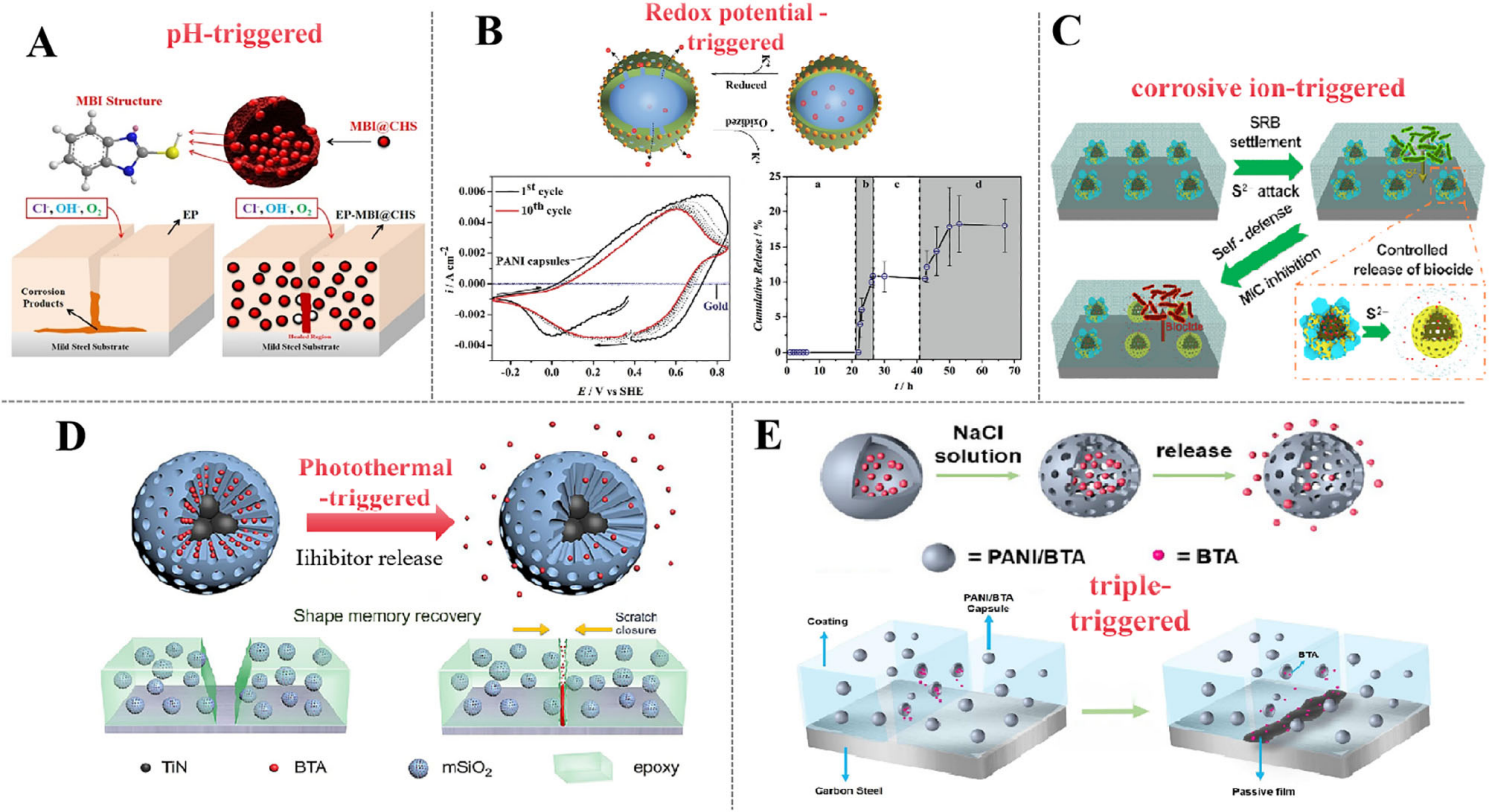
Stimulus‐responsive self‐repairing coating systems. (A) Schematic illustration of the active/self‐repairing inhibition mechanism of the EP‐MBI@CHS coating. Reproduced with permission [162]. Copyright 2021, Elsevier. (B) Cumulative release of 3‐NisA from PANI capsules under reduced and oxidized conditions and schematic representation of the release mechanism. Reproduced with permission [165]. Copyright 2013, Wiley‐VCH Verlag GmbH & Co. KGaA, Weinheim. (C) Microbiological corrosion protection mechanism for the intelligent coating. Reproduced with permission [167]. Copyright 2021, Elsevier. (D) The schematic diagram of photothermal‐triggered self‐repairing performance of epoxy resin immobilized with TiN‐BTA@mSiO_2_ NPs. Reproduced with permission [170]. Copyright 2021, Elsevier. (E) schematic diagram of the self‐repairing coating containing PANI/BTA nanocapsules. Reproduced with permission [174]. Copyright 2024, Elsevier.

Corrosion process involves pH changes in solution of localized erosion areas, thus pH changes often act as the typical stimulus for the release of corrosion inhibitors from micro/nano containers. In an early study, a hybridized zirconia (ZrO_2_)/SiO_2_ coating was prepared by encapsulating polyelectrolyte (polyethylene diimide/polyphenylene sulfonate, PEI/PSS) layers and the layers of SiO_2_ nanoparticles loaded with corrosion inhibitor (BTA) through layer‐by‐layer assembly [161]. The stimulation lies in pH changes of polyelectrolyte layered structure, leading to the decomposition of PSS/BTA complex and facilitating the release of BTA from broken vessel. Similarity‐based mechanisms, Haddadi et al. [162] prepared alkaline‐responsive self‐repairing coatings by loading corrosion inhibitor (MBI) in carbon nanorods (Figure 16A). The highest rate of MBI released from vessel was achieved at pH 11, and the corrosion inhibition layer notably prevented the metal from further corrosion. However, there are few reports on coatings that self‐repair only under acidic conditions, several studies have combined self‐repairing under acidic and alkaline conditions into one coating system [163].

Electrochemical potential differences during corrosion attack can also become triggers for stimulating containers response [164]. Polyaniline (PANI) is a typical redox‐stimulation responsive conductive polymer due to its redox performance switching between two different forms. Vimalananda et al. [165] synthesized PANI nanocapsules consisting of gold nanoparticle (AuNP) decorated in the shell and 3‐nitrosalicylic acid (3‐NISA) as corrosion inhibitor was encapsulated in the core. Figure 16B explained PANI capsules decorated with AuNPs have cyclic redox response and the reversibility of chemical‐structure changes of shell material in corrosive environment. The reduction action of corrosion increased the permeability of PANI shell layer and exhibited an accelerated release of corrosion inhibitors. On the contrary, the oxidation reaction of metal passivation decreased permeability and thus inhibited the release of corrosion inhibitors. The capsules show a striking reversibility, they can be switched to a breathable state by reduction, be closed again by applying higher potentials that correspond to passive state and re‐open again by reduction.

Ion‐exchange is a chemical process in which corrosive species can be trapped into the colonnade space of layered structures that act as scavengers for invasive species [166]. Cai et al. [167] designed novel S^2−^ responsive micro/nano containers by assembling zeolitic imidazolate framework‐8 (ZIF‐8) on the surface of hollow mesoporous silica (MSN). ZIF‐8 acted as gatekeepers to control the responsive release of loaded metronidazole corrosion inhibitors, as shown in Figure 16C. When S^2−^ concentration was more than 0.04 mm, S^2‐^ reacted with Zn^2+^ to form stable ZnS pattern, which led to the dissolution of ZIF‐8 nanovalve, thus realizing responsive release of S^2−^. Layered double hydroxides (LDH), which are composed of multilayers of actively charged mixed metallic hydroxides interspersed with anions and solvent molecules that absorb harmful chlorides and release suppressive ions [168]. Mg‐Al LDH coatings containing 8‐HQ corrosion inhibitor, which not only have the ability of ion exchange, but also have the capacity to form complexes for self‐repairing. Rationally interpret as followed that Cl^−^ was exchanged with 8‐HQ inhibitor and encapsulated in LDH containers. Afterwards, 8‐HQ was released and reacted with Mg^2+^ to form Mg(HQ)_2_ chelate layer, which restrained further corrosion and repair coating defects [169].

Light‐responsive micro/nano containers are designed primarily by adding photocatalytic materials with photosensitive groups capable of absorbing light to trigger chemical reaction, such as titanium dioxide, tungsten oxide and cadmium sulfide [170]. It has been reported that visible and IR laser irradiation to stimulate polyelectrolyte multilayer shells (PEI/PSS) or container cores (TiO_2_) modified with Ag nanoparticles (AgNPs). The metallic particles served as absorption centers to induce local heating, which disrupted the polymer shells and released BTA [171]. He et al. [172] constructed photocatalytic TiO_2_ nanostructures formed by cladding the corrosion inhibitor 8‐HQ on polyelectrolyte nanostructures, which can be inactivated by UV light to implement self‐repairing performance. The caustic aluminum surface was optically repaired by stimulating nanocontainers with UV light to release 8‐HQ. Generally speaking, light stimulation also produce thermal effect, so some researchers add polymers that can also self‐repair under thermal stimulation of photostimulation. Ma et al. [170] conducted a dual‐stimulation responsive self‐repairing coating by adding TiN‐BTA@SiO_2_ nanocontainers loaded with BTA corrosion inhibitor to shape memory epoxy coating (Figure 16D). The TiN nanoparticles under thermal effect not only promoted the release of BTA corrosion inhibitor into cracked areas under NIR irradiation, but also triggered the shape memory effect to repair the scratched areas by increasing the coating surface temperature.

Under magnetic guidance, the transmission lines of chemicals released from magnetic microcapsules can be substantially shorter than those of non‐magnetic microcapsules, which can assist corrosion inhibitors in quickly forming passivation films on damaged sites [173]. Recently, Liu et al. [174] prepared magnetically responsive self‐repairing microcapsules loaded with Fe_3_O_4_ magnetic particles and polyurethane (PU) coatings with biomimetic interfaces wet‐adhesion patterned microstructures. As a second feature that COMSOL software simulated the magnetic field design and distribution, and analyzed the magnetization phenomenon of the microstructure. The dispersion of self‐repairing microcapsules was remarkably improved under magnetization, and the adhesion of the coating is improved by the magnetic field force [175, 176]. It can be seen that the existence of magnetic fields entail stratification of individual containers. Flaxseed oil (LO) and magnetized BTA were moved into polyurea‐formaldehyde microcapsules respectively, LO microcapsules were concentrated on the upper portion of coatings by push‐out and floating effects, and BTA microcapsules were incorporated with magnetic zinc oxide, which can be migrated by magnetic gravity. In this way, it seems that magnetically response containers in coatings can be uniformly distributed under the magnetic field, directly repairing coating defects and reducing unnecessary paths [176].

In the core‐shell structures, shell polymers with different response properties can be customized to coordinate with multiple environmental stimulation. As show in Figure 16E, Huang et al. [177] synthesized PANI nanocapsules loaded with BTA inhibitor, and putted nanocapsules into shape memory polymers (SMP) epoxy coatings to realize triple‐stimuli‐responsive self‐repairing. Firstly, PANI can achieve the controllable release of BTA; Secondly, PANI can facilitate the formation of Fe_2_O_3_ passive layer; Lastly, SMP coatings can reduce the size of scratches under heated conditions, which is favorable for the closure of coating cracks. There are also substances that can change their structures in response to thermal stimulation for the preparation of responsive micro/nano containers. Recent study designed silica/polymer double‐walled hybrid nanotube composed of the multi‐stimulus responsive (pH, temperature, redox) polymer outer wall. The carboxyl groups in outer wall could control the release of BTA in response to pH changes. Poly‐*N*‐isopropylacrylamide (PNIPAM) as temperature‐responsive active molecule, this phenomenon was caused by the outer wall of PNIPAM expanded at low temperature (25°C) and shrank at high temperature (50°C), accordingly BTA were controlled to release under temperature shifts. In addition, the presence of disulfide bonds in coating matrix also promoted it to exhibit redox‐responsive release [178]. Such design allows micro/nano containers to be used not only in drugs transportation, but also for self‐repairing coatings on metals.

Intelligent combination design of structured diverse nanocontainers can provide better dispersion and compatibility with coatings as well as enhanced the charging efficiency of corrosion inhibitors, which can encompass disparate stimulus‐responsive release strategies. Via the supplementary merits of various nanocontainers, metal protective coatings can be imparted with miscellaneous self‐repairing properties and effective protection mechanisms, yet the sophisticated process of composite design also obstructs scale‐up production.

When evaluating the potential applications of self‐repairing organic coatings, it is essential to mention the automotive industry, where various commercial products are already available. Most of these products are ceramic coatings primarily composed of inorganic materials like silicon dioxide rather than polymer binders. These upmarket automotive coatings can repair small, visible cracks, such as key scratches, either through heating or by exposure to sunlight over several days. Additionally, some commercial self‐repairing coatings incorporate shape memory polymers into ceramic systems. These polymers deform when subjected to mechanical damage, like scratches, and then revert to their original shape upon heating, effectively repairing the coating.

## 5 Combination of Self‐Warning and Self‐Repairing Performance

Just as human skin, it is the first protective barrier of organism and has self‐warning function to denote injury caused by abrasion, at the same time repairing the wound autonomously [179, 180]. Coatings that act as protective barriers for metals should also similarly embody both self‐warning and self‐repairing functions. As described in the above chapters, researches focus on either self‐warning coatings or self‐repairing coatings have achieved notable advancements in the field of metal corrosion protection [181, 182], but how to simultaneously admonish damage/corrosion and renovate defects in a coating system remain problematic issues. The implementation of dual self‐warning and self‐repairing functions enables timely corrosion alerts and effective containment of corrosion activities prior to manual repair, which can prevent further damages to metal substrates and prolong the service life of both coatings and metals. Obviously, self‐warning and self‐repairing capabilities are complementary to collaboratively augment the comprehensive performance of coatings [38, 49].

To realize both self‐repairing and self‐warning functions in a coating system, then it is required to add materials with both functions to the coating matrix. Currently, the types of materials that exhibit both self‐repairing and self‐warning properties are exceedingly rare. Generally, they are combined with metal ions to form chelates with strong colors to enable both functionalities simultaneously. However, such single‐component coating systems, which involve the addition of only one type of material, are only suitable for specific metal surfaces. Multi‐functional coatings with a wider range of applications realize self‐repairing and self‐warning, respectively, by adding at least two substances to the coating matrix. Integrating both self‐repairing and self‐warning agents into a single coating system is considerably complex and may compromise the inherent properties of the coating. Furthermore, the choices of active sensors that offer both self‐repairing and self‐warning functionality are extremely limited, let alone optimizing their performance.

### 5.1 Single‐Component Coating Systems

**FIGURE 17:**
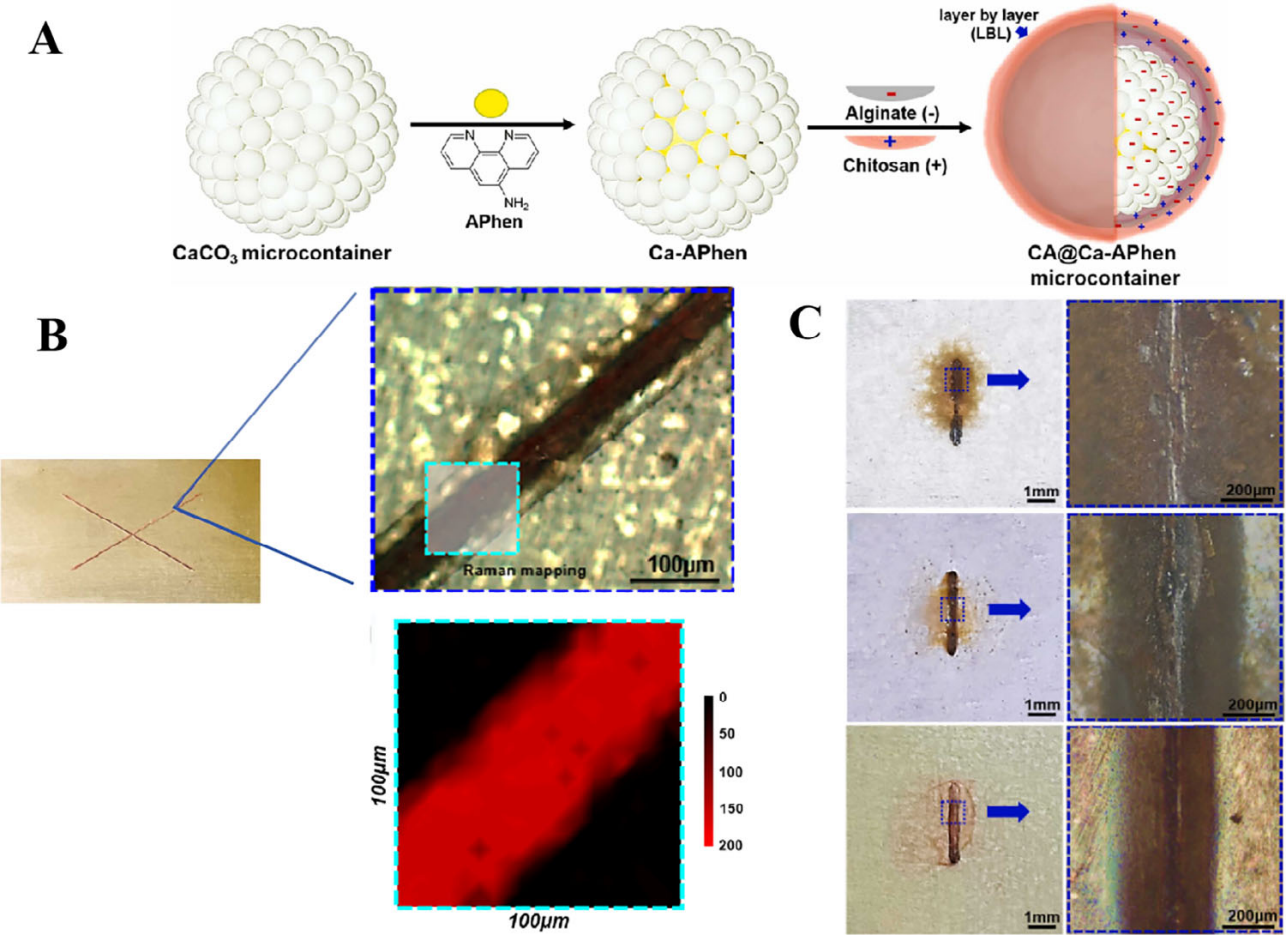
Achieving dual‐function with single‐component material. (A) Schematic illustration of the CA@Ca‐APhen microcontainer. (B) Optical images of the scratched CA@Ca‐APhen5 wt%/EP coating and Raman intensity mapping for Fe^2+^—APhen complex. (C) Optical images of the scratched neat epoxy coating, CA@Ca5 wt%/EP coating and CA@Ca‐APhen5 wt%/EP coating after 144 h of immersion in 3.5 wt% NaCl solution. Reproduced with permission [184]. Copyright 2022, Elsevier.

The simultaneous addition of two functional ingredients into coating matrix concurrently not only increases the complexity of coatings composition, but also reduces the densification of coatings, thereby weakening their barrier properties. It is a terrific research idea to add only one functional material in the coating, which can minimize the addition of components, as well as ensure the realization of dual‐function. This kind of research has several more classic cases on steel surface, especially, 1,10‐phenanthroline (Phen) and its derivatives are color indicators for detecting Fe^2+^, which are thoroughly mentioned in chapter 2 of metal ion indicators [34, 50]. Beyond, adding Phen or its derivatives in coating matrix accomplishes dual tasks at the same time, including realizing self‐warning and self‐repairing properties in corroded areas [183]. This conclusion was confirmed by Liu [184], who designed CaCO_3_ micrometer containers loaded with 1,10‐phenanthroline‐5‐amine (APhen) and added them into epoxy coating (Figure 17A). The damaged regions of coating exhibited red color within only 2 min as early warning of corrosion onset and the intensity of color significantly increased during 1 h of salt spray testing, which could showed strong signal of the Fe^2+^‐APhen complex along the scratch after only 2 min (Figure 17B). CA@Ca‐APhen5 wt%/EP coating showed relatively little rust in and around the scratch after 144 h of immersion, indicating corrosion on the exposed steel substrate was well inhibited (Figure 17C). The rapid localization of early corrosion by the coating is not only vital for timely corrosion warning in practical applications, but also for self‐repairing of the coating as well as manual maintenance at later stage.

More recently, tannic acid (TA) has also been confirmed to chelate with Fe^3+^ to produce vibrant color changes, because of the abundant o‐phenylphenol moiety inside its structure [185]. MSN‐TA (TA was loaded into mesoporous silica nanocontainer) to synthesized the multifunctional (weather‐resistant, self‐repairing, and self‐warning) reactive epoxy coating, it showed that an evident black line was generated along the scratch of the composite coating after 5 h of spraying and revealed an obvious increase of high‐frequency phase angles with immersion time, demonstrated its significant self‐warning and self‐repairing effects [186]. The evolution of corrosion self‐warning and self‐repairing properties are two vital dimensions for extending the service life of protective coatings and underlying metal structures.

8‐hydroxyquinoline (8‐HQ) is also chronicled in second chapter, based on its specialty that can form fluorescent probes with various metal ions to warn of corrosion initiation [51, 187]. In addition to restorative capability, as the second bonus characteristic, an in‐depth reason to explain the repair effect is that its strong coordination by non‐bonded electrons of oxygen and nitrogen with metal surface. Some experimental works implemented by Hu et al. [188] which revealed that dissolved Al^3+^ ions in corroded Al substrates displace Ce^3+^ ions in the Ce‐HQ complex, forming a fluorescent Al‐HQ indicative of coating flaws. In addition, the generated Al‐HQ constructed protective layers on the substrate to inhibit corrosion, and the released Ce^3+^ also prevented the reduction of O_2_ on the Al substrate to form CeO_2_ deposition. Single material can simultaneously emit the self‐warning and self‐repairing functions, which not only simplifies the coating preparation process, but also provides timely warning and repairing in the same environment, thereby extends the coating service life and reduces the cost of manual maintenance. Throughout the whole process, it seems that in direction of the advancement of dual‐functional coatings, reagents with double functions ought to be prioritized first.

In single‐component coating systems, achieving both self‐warning and self‐repairing functions simultaneously typically involves adding both functions to the same microcapsule system. Simply encapsulating the single‐component functional material eliminates the need for manually adding other triggering agents. However, this approach significantly limits repair efficiency. Currently, it is mainly used on the surfaces of steel and aluminum alloys.

### 5.2 Multi‐Component Coating Systems

Currently, the classification of dual‐functional coatings remains under development, with a proposed approach involving the encapsulation of fluorescent/color indicators and corrosion inhibitors within containers to be integrated into a singular coating matrix, provisionally classified as multi‐component system [189]. It is reported that the application of materializing self‐warning and self‐repairing functions uniformly only rarely cases, and the scope of researches is somewhat limited. To broaden the availability of dual‐functional coatings, a variety of combinations involving the incorporation of both warning and repairing functional materials into a single coating matrix are being explored. This strategy ensures that both functionalities are activated in response to the same corrosive stimuli, potentially broadening the scope and effectiveness of dual‐functional coatings [190, 191, 192].

A number of parameters should be taken into account, such as the choice of micro/nano containers, corrosion indicators, and corrosion inhibitors/repair agents matched in one coating matrix. In other words, the best warning indicators and repair agents can be combined with each other to optimize coating performance as far as possible, without being limited to the inherent warning and repair ability of single material. In an earlier study, Wang et al. [193] synthesized a class of PDVB‐graft‐P(DVB‐co‐AA) core–shell microspheres, specialized porosity allowed for encapsulation of corrosion inhibitor BTA and fluorescent probe coumarin. The polymer coatings could respond to the elevated pH during corrosion reaction by embedding two microspheres. Under alkaline conditions, green fluorescent signals exhibited near the cracks could be explicitly observed under UV light, at the same time BTA were released to complex with metal cations to cover corroded areas.

Depends on variegated duty circumstances of the coatings, it is necessary to adjust counterpart response mechanisms so that broaden the applicability range of dual‐functional coatings. A detailed discussion of stimulation mechanism that two saline‐responsive polyaniline (PANI) nanocapsules, including nanocapsules containing fluorescein isothiocyanate (FITC) as fluorescent indicator and benzotriazidazole (BTA) as corrosion inhibitor. Na^+^ react with the surface of PANI capsules in the interstitial space after coating was cracked, leading to the gating effect of both nanocapsules to release FITC and BTA. One microscopic reason for this is the forces between molecular chains, in salt solution, the polyaniline molecular chains are adulterated by NaCl, resulting in the hydrogen bonding forces are weakened and causing the stacking between polymer chains to become loose. Then, the rigidity of the polyaniline molecules were decreased, leading to consequential phase transition. In addition, after salt doping, the electronic structure of the polymer chains were changed, which also made it more hydrophilic and the hydrophilic substances more prone to osmosis [194].

Owing to the difficulty of integrating different functional elements and lacking of suitable containers for host triggers with response characteristics. Apart from mixing two functional containers into one coating, sometimes the coating matrix itself can also act as material with self‐repairing properties. It has been suggested that epoxy resin can behave healing effect with appropriate catalysts. A comprehensive use of flexible reagents that the pioneer chose to incorporate the triggering agent 25 wt% of tetraethylpentylamine with 75 wt% of JEFFAMINE T403 (25TEPA75T403) and 2',7'‐dihydrodichlorofluorescein (DCF) as pH indicator into microcapsules. 25TEPA75T403 not only cured the coating matrix epoxy blend (F10B) with high performance to fully restore type I fracture toughness of the coating, but also allowed F10B‐DCF to display strong red warning signals. The warning signals were highly visualized to distinguish the damages of coating, and more importantly, the self‐repairing rate was up to 100%. It is worth noting that this straightforward procedure, with entirely autonomous warning and repairing capabilities independent of the host matrix [195]. Furthermore, there are some measurements on the coating system contain three multifunctional components. CVL, epoxy monomer, and phenyl acetate were encapsulated in one silica nanoparticle (SNs), SNs and epoxy curing agents were separately dispersed in waterborne epoxy resins (WEP) substrates. The CVL and SNs showed intense blue color to warn of coating damages, and the epoxy monomer (2020A) and curing agent (2020B) played a noteworthy role in self‐repairing reaction up to 92.92% [196]. When multiple substances were intermingled in the same coating matrix, it should be regarded that the use of visual warning indicator, a conspicuous substance with significant contrast to the color of coating surface can be elected for an accurate representation of damages.

**FIGURE 18:**
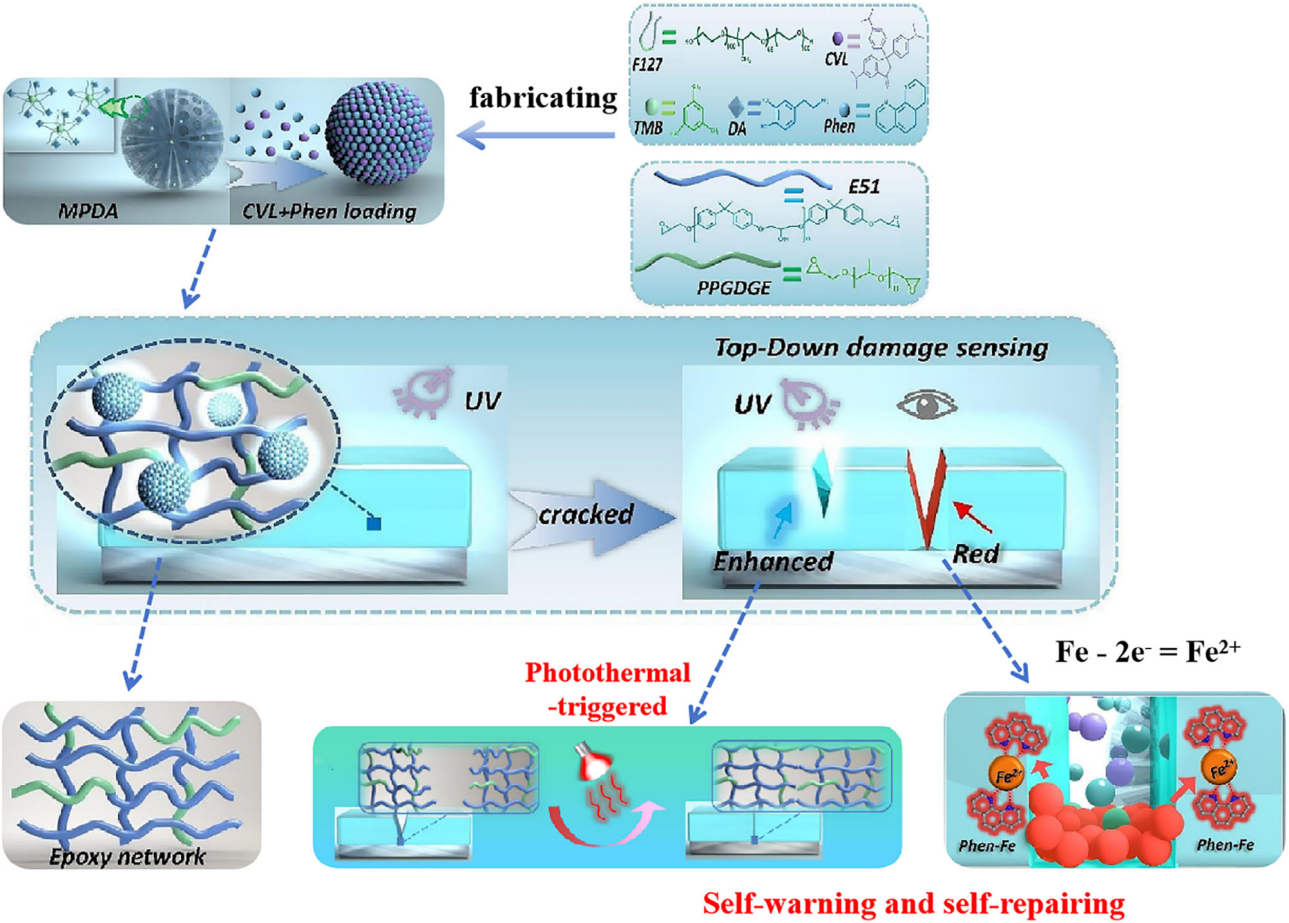
Schematic illustration of fabricating the hierarchically self‐reporting and self‐repairing coating. Reproduced with permission [197]. Copyright 2023, Elsevier.

However, which is the most important? Warning of coatings damages or metal corrosion? For conventional anti‐corrosion coatings, it is a challenge to simultaneously warn of two failure stages involving coating damages and corrosion attack, additionally to restore the barrier performance of coatings while mitigating the metal substrate corrosion. Then Cheng et al. made some ingenious attempts [197], they added mesoporous dopamine nanoparticles (MPDA) loaded with both CVL and Phen into crosslinked thermoresponsive epoxy resin coating (Figure 18). CVL exhibited a blue luminescence to warn of coating damages. In addition, Phen molecule acted as corrosion indicator complexing with Fe^2+^ generated from localized corrosion to produce an intense red color representing the early warning signals of corrosion attack. The two different luminescence and color change mechanisms assigned the coating the graded self‐warning function. Utilizing photothermal conversion agent (MPDA), the thermosensitive resin had the effect of rapidly repairing coating cracks under 90 s NIR irradiation, and Phen‐Fe^2+^ complex could also play a role in preventing further corrosion. The designed coating had achieved hierarchically self‐warning and self‐repairing functions, which had promising potential in the application of intelligent dual‐functional coatings. The simultaneous realization of early warning signals and achievement of hierarchically self‐repairing function at the coating/metal interfaces, which can continuously monitor and extend the whole service lifetime of coating/metal systems.

## 6 Future Outlook

### 6.1 Self‐Warning Coatings

The incorporation of corrosion indicators into self‐warning coatings facilitates early visual warning of corrosion and is suitable to practical industrial situations. However, the option for corrosion indicators remains limited. Specifically, for metal ion indicators, only Phen and its derivatives have been documented to react with Fe^2+^, whereas no chromatic reactions have been reported with other metal ions like Al^3+^ and Mg^2+^. There is substantial value in developing new indicators sensitive to a broader range of corrosion‐induced environmental variations. To prevent premature fluorescence signals leakage from the dyes, materials capable of UV‐light screening or fluorescence quenching may be incorporated during the encapsulation process, ensuring that only coating damage or metal corrosion acts as the fluorescence emission trigger. Furthermore, in selecting visual warning indicators, consideration should be given to the necessity of achieving a stark contrast with the color of coating surface. Substances with a strong visual impact must be selected to accurately signal damage, thereby enhancing the efficacy and precision of self‐warning functionality. This approach is both effective and accurate in ensuring that the self‐alarm function of the coating system, contributing to the overall performance and reliability of protective coatings in monitoring and signaling integrity breaches.

### 6.2 Self‐Repairing Coatings

Ideally, intrinsic self‐repairing coatings have long‐term repair capability in the presence of sufficient external energy (heat, light). Regrettably, there is a contradiction between the mechanical strength and the restorative capacity of coatings, with mechanical properties tending to diminish with successive repairs. Consequently, it is advocated to design polymers featuring multi‐phase systems that incorporate diverse chemical bonds, thus furnishing the coatings with both robust mechanical strength and superior self‐repairing capabilities. Conversely, extrinsic self‐repairing coatings typically provide only limited, sometimes single‐use, repair opportunities, thereby undermining the sustainability of coating's functionality. For practical implementation, synthesizing the methodologies of intrinsic and extrinsic mechanisms presents a pragmatic strategy, enhancing the lifetime and effectiveness of self‐repairing coatings in practical applications.

### 6.3 Dual‐Functional Coatings

With regard to the addition of microcapsules, an excess can lead to their agglomeration, and ultimately to conspicuous defects at the capsule/coating interface. Within the framework of achieving dual‐function, the compositional simplicity of coatings correlates with a denser system architecture, underscoring the necessity to harness multifunctional chemicals capable of concurrently detecting and remedying coating damages, exemplified by agents such as Phen and 8‐HQ. Nonetheless, the imperative of maintaining barrier protection and densification within coatings should not be overlooked, as it is critical to establish a dynamic balance among the diverse functionalities of coatings. To enhance coating performance, refining the encapsulation technology is essential, focusing on precisely controlling over the size of containers and ensuring that it is uniformly distributed within coating matrix, thereby optimizing the multifaceted capabilities of dual‐functional coatings.

## 7 Conclusion

In conclusion, functional coatings possess vast application prospects owing to their self‐warning, self‐repairing and dual‐functional characteristics in the field of metal corrosion and protection. This review has endeavored to comprehensively cover all extant indicators related to self‐warning coatings, which include corrosion indicators that exhibit pronounced fluorescent signals or color changes in response to environmental changes (such as metal ions and pH) and mechanically triggered indicators that warning of coating damage, with an emphasis on the fluorescence/coloration performance of various coatings. Meantime, the advancements in self‐repairing coatings have been summarized based on means of implementation: intrinsic and extrinsic self‐repairing, with the aspiration of presenting classic examples that illuminate current trends and prospective design opportunities. Looking forward, self‐repairing coatings should be further optimized through the combination of two methods in a singular system. It is also pointed out that dual‐functional coatings are categorized as single‐component systems and multi‐component systems, underscoring their significant promise for achieving harmonious balance of functionalities and technical refinement. This review aims to provide strategic guidance for prudent design and fabrication of sustainable functional coatings, and to point future research directions in this dynamic and evolving field.

## Conflicts of Interest

The authors declare no conflicts of interest.
